# Deletion of p53-Related Protein Kinase Suppresses Solar UV–Induced Photocarcinogenesis by Inhibiting PD-L1 Expression and Enhancing CD8 T-Cell Infiltration

**DOI:** 10.1016/j.jid.2025.07.021

**Published:** 2025-08-12

**Authors:** Qiushi Wang, Eunmiri Roh, Asad U. Khan, Sally E. Dickinson, Georg T. Wondrak, Ann M. Bode, Clara Curiel-Lewandrowski, Tianshun Zhang

**Affiliations:** 1 The Hormel Institute, University of Minnesota, Austin, Minnesota, USA; 2 Department of Cosmetic Science, Kwangju Women’s University, Gwangju, Republic of Korea; 3 The University of Arizona Cancer Center, The University of Arizona, Tucson, Arizona, USA; 4 Division of Dermatology, College of Medicine-Tucson, Tucson, Arizona, USA

**Keywords:** CD8 T-cell infiltration, PD-L1, PRPK, SUV-induced skin cancer

## Abstract

Nonmelanoma skin cancers are primarily caused by solar UV exposure and represent the most common cancers in the United States. PRPK (p53-related protein kinase) is a protein kinase that is involved in multiple cancers, including colon cancer, myeloma, and hepatocellular carcinoma. In this study, we generated epidermal-specific PRPK-knockout mice using CRISPR/Cas9 technology in SKH1 hairless mice with loxP-flanked PRPK alleles, crossed with keratin 14-Cre (K14.Cre) mice. Our findings reveal that epidermal-specific deletion of PRPK significantly suppresses tumor growth in solar-simulated light–induced nonmelanoma skin cancer. Knocking down PRPK significantly suppresses cutaneous squamous cell carcinoma cell growth by inducing G1 phase arrest and promoting apoptosis. Mechanistically, PRPK deletion inhibits proliferating cell nuclear antigen and PD-L1 expression as well as the expression of transcription factors c-Myc, c-Jun, NF-κB, and activator protein-1, which mediate PD-L1 expression. Using a 3-dimensional culture system, we further demonstrate that PRPK deletion suppresses cutaneous squamous cell carcinoma cell growth. Flow cytometry analysis indicates that PRPK deletion enhances CD8 T-cell infiltration. This is accompanied by significant reductions in IL-6, MIP-2, and VEGF levels, reprogramming the tumor microenvironment to support CD8 T-cell infiltration. In summary, our study demonstrates that PRPK deletion suppresses solar UV–induced photocarcinogenesis by inhibiting PD-L1 expression and enhancing CD8 T-cell infiltration, highlighting its potential as a therapeutic target for nonmelanoma skin cancer.

## INTRODUCTION

Nonmelanoma skin cancers (NMSCs), including cutaneous squamous cell carcinoma (cSCC), are the most common malignancies in the United States and pose a significant public health challenge (Aggarwal et al, 2021; Rogers et al, 2021). Chronic exposure to solar UVR is the primary cause of NMSC, triggering a series of molecular events, such as DNA damage, various kinase responses, and immune modulation (Ciążyńska et al, 2021; Gao et al, 2017; Lee et al, 2020; López-Camarillo et al, 2012). These processes drive tumor development and foster an immunosuppressive tumor microenvironment (TME) that impairs effective antitumor immune responses. Despite the rising prevalence of NMSC, preventive measures, such as sunscreen use and public education on UV protection, have not significantly reduced its incidence. In addition, chemoprevention options remain limited, with available agents showing variable response/recurrent rates and local toxicity, leading to compliance barriers that lower the threat in high-risk populations (Sol et al, 2024). These challenges underscore the urgent need to identify molecular targets and mechanisms to enhance prevention and treatment strategies for NMSC.

Immunosuppressive TME is a critical hallmark of cancer progression (Hosseini et al, 2024; Mortezaee, 2020). One characterization of an immunosuppressed TME is diminished infiltration and impaired activation of cytotoxic T cells, alongside elevated expression of immunosuppressive molecules such as PD-L1 (Ai et al, 2020; Mousa et al, 2024; Zhang et al, 2020). This hostile environment fosters immune evasion by tumors, suppressing antitumor immunity and facilitating cancer growth and metastasis (Haynes et al, 2024; Kim and Cho, 2022). Identifying the molecular regulators that influence both tumor cell behavior and the TME is crucial for advancing NMSC prevention and treatment approaches.

PRPK (p53-related protein kinase, also known as TP53RK) is a serine/threonine kinase that has emerged as a potential oncogenic factor in various cancers, including colon cancer, multiple myeloma, and hepatocellular carcinoma (Dwira et al, 2024; Hideshima et al, 2017; Zykova et al, 2018, 2017). PRPK is a component of the KEOPS (kinase, endopeptidase, and other proteins of small size) complex, which is involved in transfer RNA modification (Beenstock et al, 2020), but its roles extend beyond translational regulation to influence cell proliferation, survival, and stress responses (Hideshima et al, 2017; Peterson et al, 2010; Zykova et al, 2018). Our previous study demonstrated that PRPK functions as a downstream effector of TOPK (T-LAK cell-originated protein kinase), a key kinase involved in cSCC progression (Gao et al, 2017; Roh et al, 2023, 2020, 2018). Suppressing PRPK activation with its inhibitor, rocuronium bromide, effectively attenuated solar simulated light (SSL)–induced photocarcinogenesis in mouse models, suggesting its potential as a therapeutic target in cancer prevention (Roh et al, 2018). Despite these associations, the role of PRPK in skin cancer, particularly in UV-induced photocarcinogenesis, remains unclear.

In this study, we demonstrate the function of PRPK in both tumor growth and the immune environment, which offers an opportunity to develop dual-action therapies capable of addressing both the intrinsic drivers of cancer and the extrinsic immunosuppressive environment. Keratinocytes are known for their dual role as both epithelial and immune cells (Simmons and Gallo, 2024). This dual approach is particularly strategic in the aging population because immunosenescence and the increased rate of multiple primary NMSC represent an important healthcare challenge. This study focuses on elucidating the role of PRPK in solar UV–induced skin carcinogenesis to identify potential avenues for therapeutic intervention in NMSCs.

## RESULTS

### Skin-specific knockout of PRPK efficiently blocks SSL-induced skin cancer

**Figure 1.**
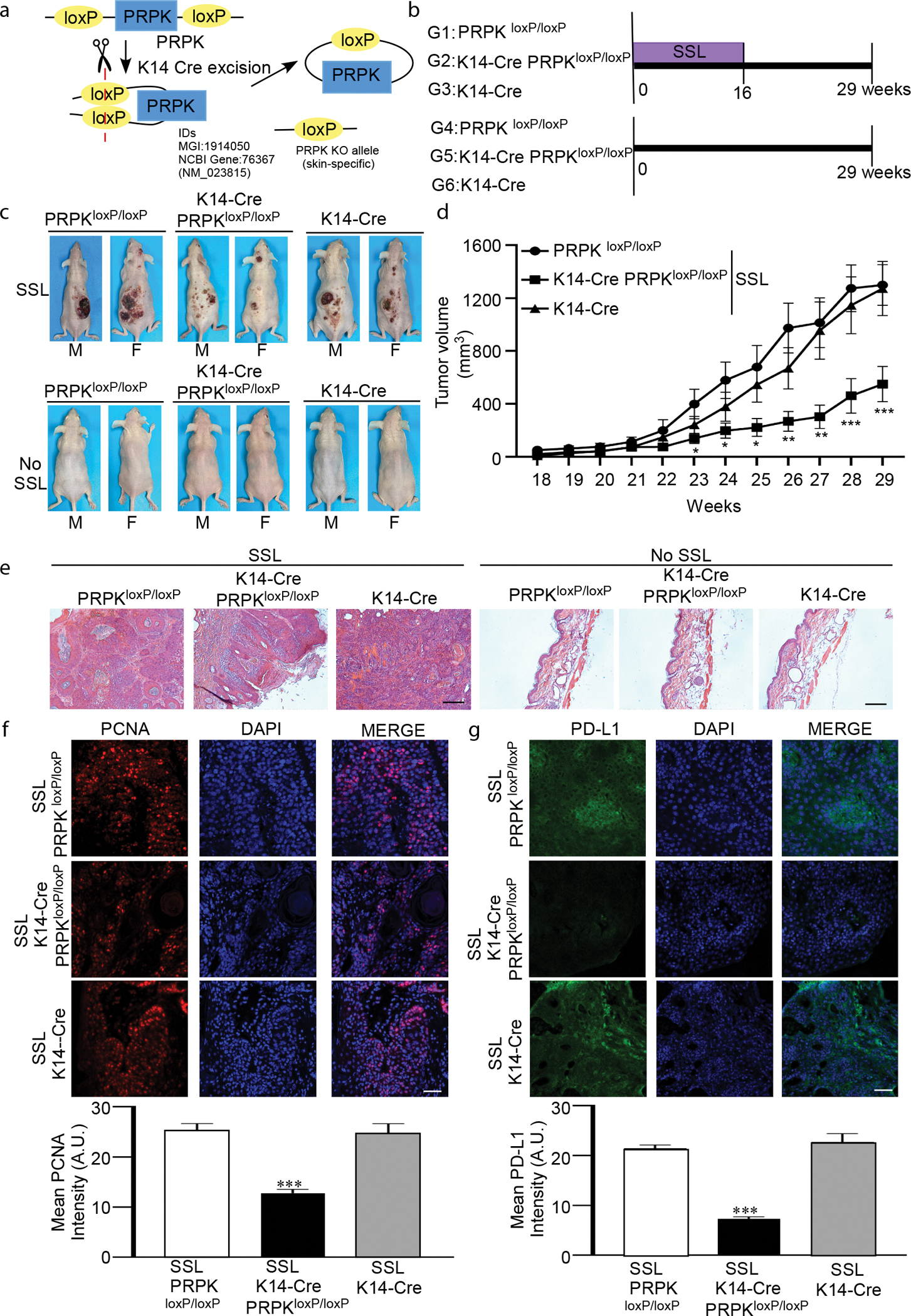
Deletion of PRPK in the skin epidermis efficiently suppresses SSL-induced cSCC in a mouse model. **(a)** CRISPR/Cas9 genome engineering was used to generate floxed PRPK mice (PRPK^loxP/loxP^). To create skin epidermis–specific PRPK-knockout mice, PRPK^loxP/loxP^ SKH-1 mice were crossed with K14-Cre mice. (**b)** The experimental design for SSL-induced photocarcinogenesis model using PRPK^loxP/loxP^, PRPK^loxP/loxP^ K14-Cre, and K14 mice (groups 1–3). Control mice were not treated with SSL (groups 4–6). (**c)** Conditional PRPK-knockout mice (K14-Cre, PRPK^loxP/loxP^) exhibited markedly reduced SSL-induced tumor growth compared with control groups (PRPK^loxP/loxP^ or K14). **(d)** Tumor volume was calculated using the following formula: tumor volume (mm^3^) = length × width × width × 0.52. Data are presented as mean values ± SD, and statistical differences were determined by 1-way ANOVA. The asterisks indicate a significant decrease in tumor growth compared with that in control groups exposed to SSL or treated with a vehicle followed by SSL exposure (*P < .05, **P < .01, and ***P < .001). (**e)** Histopathological differences between groups were assessed using H&E staining (bar = 100 μm). **(f, g)** IF analysis was performed to assess the levels of PCNA and PD-L1 in tumors from K14-Cre, PRPK^loxP/loxP^ mice and control groups (PRPK^loxP/loxP^ or K14) with SSL irradiation. Signal intensity was quantified using the ZEISS ZEN 3.7 program (lower panels). Bar = 50 μm. The asterisks (***) denote a significant decrease (*P* < .001) in the K14-Cre, PRPK^loxP/loxP^ group compared with the PRPK^loxP/loxP^ and K14-Cre groups. A.U., arbitrary unit; cSCC, cutaneous squamous cell carcinoma; F, female; IF, immunofluorescence; M, male; PCNA, proliferating cell nuclear antigen; SSL, solar simulated light.

To investigate the role of PRPK in solar UV–induced skin carcinogenesis, we utilized the CRISPR/Cas9 system to establish PRPK^loxP/loxP^ SKH1-Hr^hr^ mice at the University of Arizona Genetically Engineered Mouse Models Core. To generate skin epidermis–specific PRPK knockout mice, the PRPK ^loxP/loxP^ SKH-1 mice were crossed with K14-Cre mice (Figure 1a). The results of PRPK ^loxP/loxP^, K14-Cre PRPK^loxP/loxP^, and K14-Cre SKH-1 mice were identified (Supplementary Figure S1). Then, we examined chronic SSL-induced skin carcinogenesis in epidermis-specific knockout (K14-Cre PRPK^loxP/loxP^) and control hairless (PRPK^loxP/loxP^ and K14-Cre SKH1-Hr^hr^) mice (Figure 1b). Notably, PRPK epidermisspecific knockout (K14-Cre PRPK^loxP/loxP^) mice exhibited significantly suppressed tumor growth compared with the control groups after SSL exposure. No tumors were observed in any group of mice without SSL exposure (Figure 1c and d). Histopathological differences between groups were assessed using H&E staining (Figure 1e). In addition, the proliferation marker, proliferating cell nuclear antigen, was markedly reduced in the skin of SSL-exposed K14-Cre PRPK^loxP/loxP^ mice compared with that in the control groups (Figure 1f). Furthermore, levels of PD-L1 were significantly decreased in the epidermis-specific knockout mice (K14-Cre PRPK^loxP/loxP^) with SSL treatment compared with that in SSL-exposed control groups (Figure 1g). No significant differences in proliferating cell nuclear antigen or PD-L1 expression were observed between groups without SSL exposure (Supplementary Figure S2). These findings suggest that PRPK plays a crucial role in solar UV–induced skin carcinogenesis and modulates the immune checkpoint PD-L1 in response to SSL exposure.

### PRPK knockdown suppresses proliferation, induces G1 cell cycle arrest, and promotes apoptosis in cSCC cells

**Figure 2.**
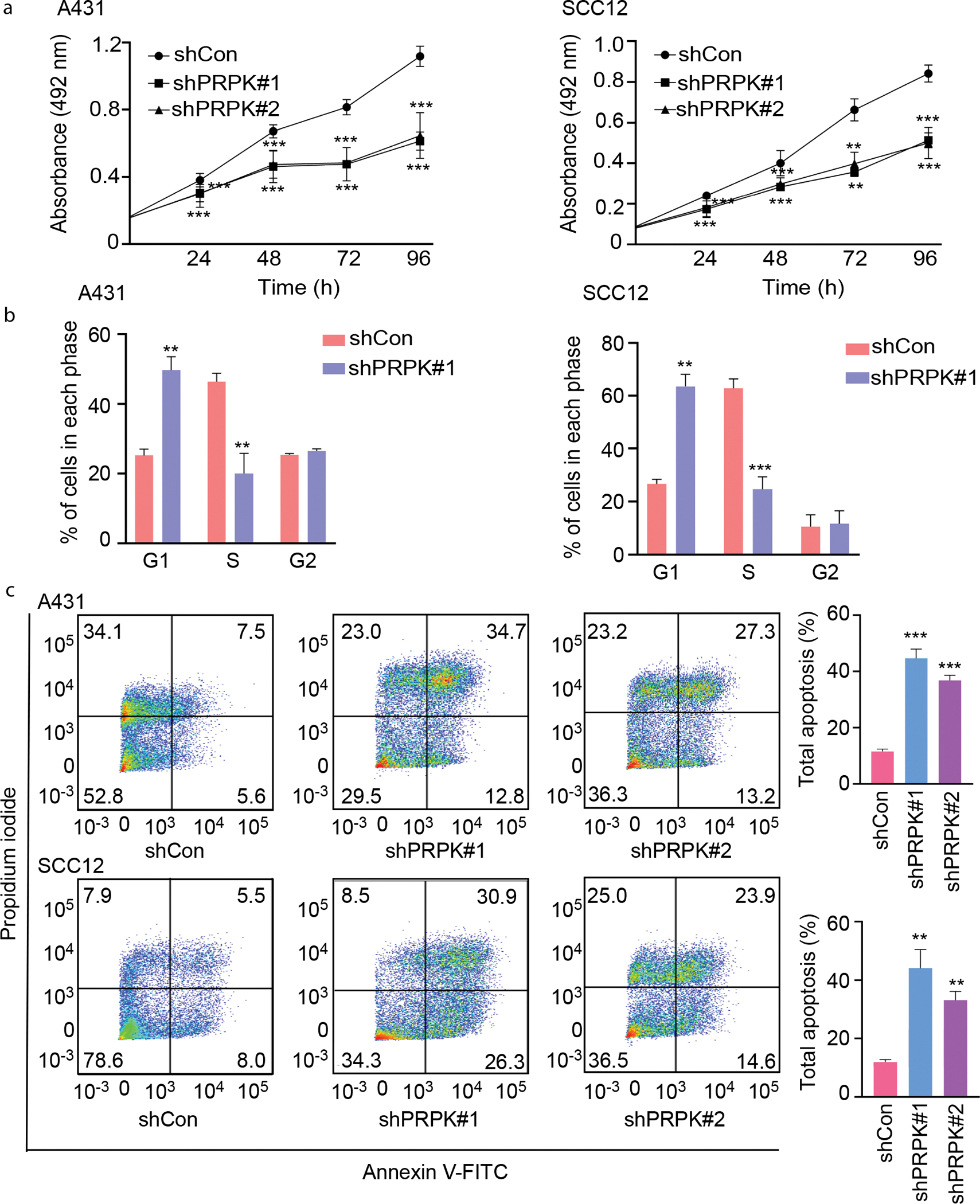
Knockdown of PRPK suppresses proliferation, induces G1 arrest, and promotes apoptosis in A431 and SCC12 cells. (**a)** Cell viability was measured using MTT assays in A431 and SCC12 cells transduced with shCon or 2 independent shRNAs targeting PRPK (shPRPK#1 and shPRPK#2). Knockdown of PRPK significantly suppressed cell growth over time. Data represent mean ± SE of 3 independent experiments. (**b)** Cell cycle distribution was analyzed by flow cytometry after PI staining in A431 and SCC12 cells with or without PRPK knockdown. PRPK depletion led to a significant increase in G1 phase and a decrease in S phase populations, indicating G1 phase arrest. (**c)** Apoptosis was assessed by Annexin V-FITC/PI double staining and flow cytometry. PRPK knockdown markedly increased the percentage of apoptotic cells in both A431 and SCC12 cell lines. Representative dot plots are shown along with quantification of total apoptosis (early + late). Data shown as mean ± SE (n = 3). Statistical analysis was performed using 1-way ANOVA. **P < .01 and ***P < .001 versus shCon. h, hour; PI, propidium iodide; SE, standard error; shCon, control-targeted short hairpin RNA; shPRPK, PRPK-targeted short hairpin RNA; shRNA, short hairpin RNA.

To determine the functional role of PRPK in cSCC cell growth, we performed loss-of-function studies using 2 independent short hairpin RNAs targeting PRPK in A431 and SCC12 cell lines. MTT assays revealed that PRPK knockdown significantly reduced cell viability over time compared with that in control cells (Figure 2a), indicating that PRPK is critical for cSCC cell proliferation. Next, we evaluated the effect of PRPK knockdown on cell cycle progression. Flow cytometric analysis after propidium iodide (PI) staining showed a marked increase in the G1 phase population and a concomitant reduction in the S phase population in both A431 and SCC12 cells upon PRPK silencing (Figure 2b), suggesting that PRPK promotes G1–S cell cycle transition. Furthermore, apoptosis analysis using Annexin V-FITC/PI staining demonstrated a significant increase in the percentage of apoptotic cells in PRPK-deficient A431 and SCC12 cells compared with that in controls (Figure 2c). These findings indicate that PRPK not only supports cell proliferation but also helps to suppress apoptosis in cSCC cells.

### Deletion of PRPK suppresses PD-L1 expression by inhibiting the activation or expression of transcription factors NF-κB, c-Jun, c-Myc, and activator protein 1

**Figure 3.**
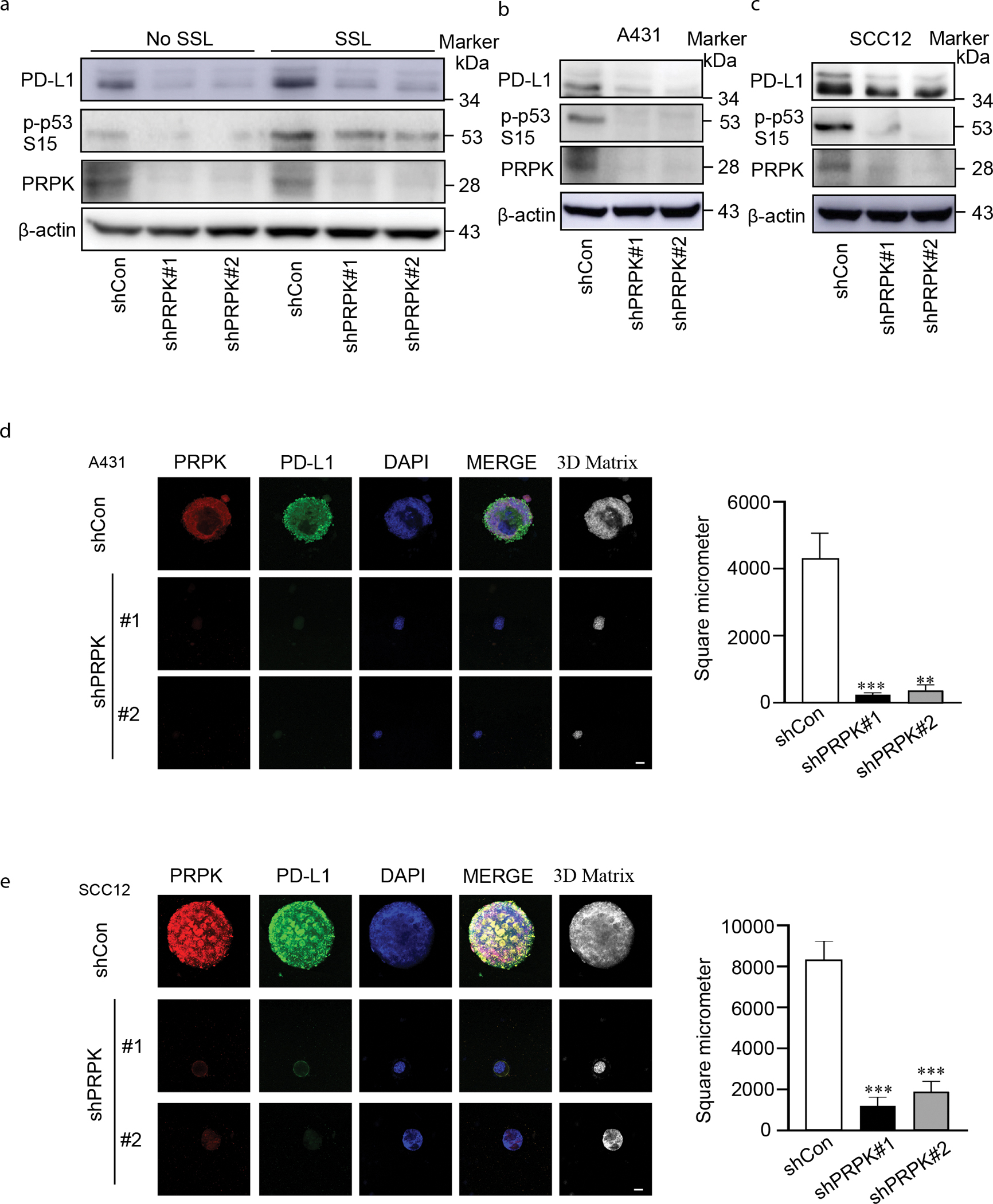
PRPK knockdown suppresses PD-L1 expression in keratinocytes and cSCC cells and inhibits cSCC cell growth. **(a)** Knockdown of PRPK reduces PD-L1 expression levels in keratinocytes with or without SSL irradiation as determined by western blotting. **(b, c)** PRPK knockdown substantially decreases PD-L1 expression in A431 and SCC12 cSCC cells. **(d, e)** PRPK knockdown inhibits A431 and SCC12 cell growth in a 3D multicellular spheroid culture system. IF analysis of 3D multicellular spheroids reveals reduced PD-L1 expression in A431 and SCC12 cells after PRPK knockdown. PRPK is shown in red, PD-L1 in green, and nuclei are counterstained with DAPI (blue). Bar =10 μm. Statistical significance was determined by 1-way ANOVA (***P < .001). 3D, 3-dimensional; IF, immunofluorescence; shCon, control-targeted short hairpin RNA; shPRPK, PRPK-targeted short hairpin RNA; shRNA, short hairpin RNA; SSL, solar simulated light.

The expression level of PD-L1 is elevated in SSL-exposed keratinocyte HaCaT cells. Knockdown of PRPK significantly reduces its downstream phosphorylated p53 at ser15 and PD-L1 expression in HaCaT cells, both with and without SSL exposure (Figure 3a). Furthermore, PRPK knockdown markedly decreases phosphorylated p53 at ser15 and PD-L1 expression in A431 and SCC12 cSCC cell lines (Figure 3b and c). In addition, 7,12-dimethylbenz[a]anthracene, a carcinogen that serves as a tumor initiator, inducing skin cancer (Nassar et al, 2015), can also increase the expression of PD-L1 and phosphorylated p53 (Ser15). The knockdown of PRPK significantly suppresses 7,12-dimethylbenz[a]anthracene–induced PD-L1 and p53 Ser15 expression in keratinocytes (Supplementary Figure S3). To further investigate the effect of PRPK on cSCC cell growth and PD-L1 expression, we utilized an immunofluorescence (IF)-based 3-dimensional culture system. Notably, PRPK knockdown significantly suppresses cSCC cell growth while concurrently reducing PD-L1 expression (Figure 3d and e). To identify the mechanism, we focused on the effect of PRPK on transcription factors mediating PD-L1 expression.

**Figure 4.**
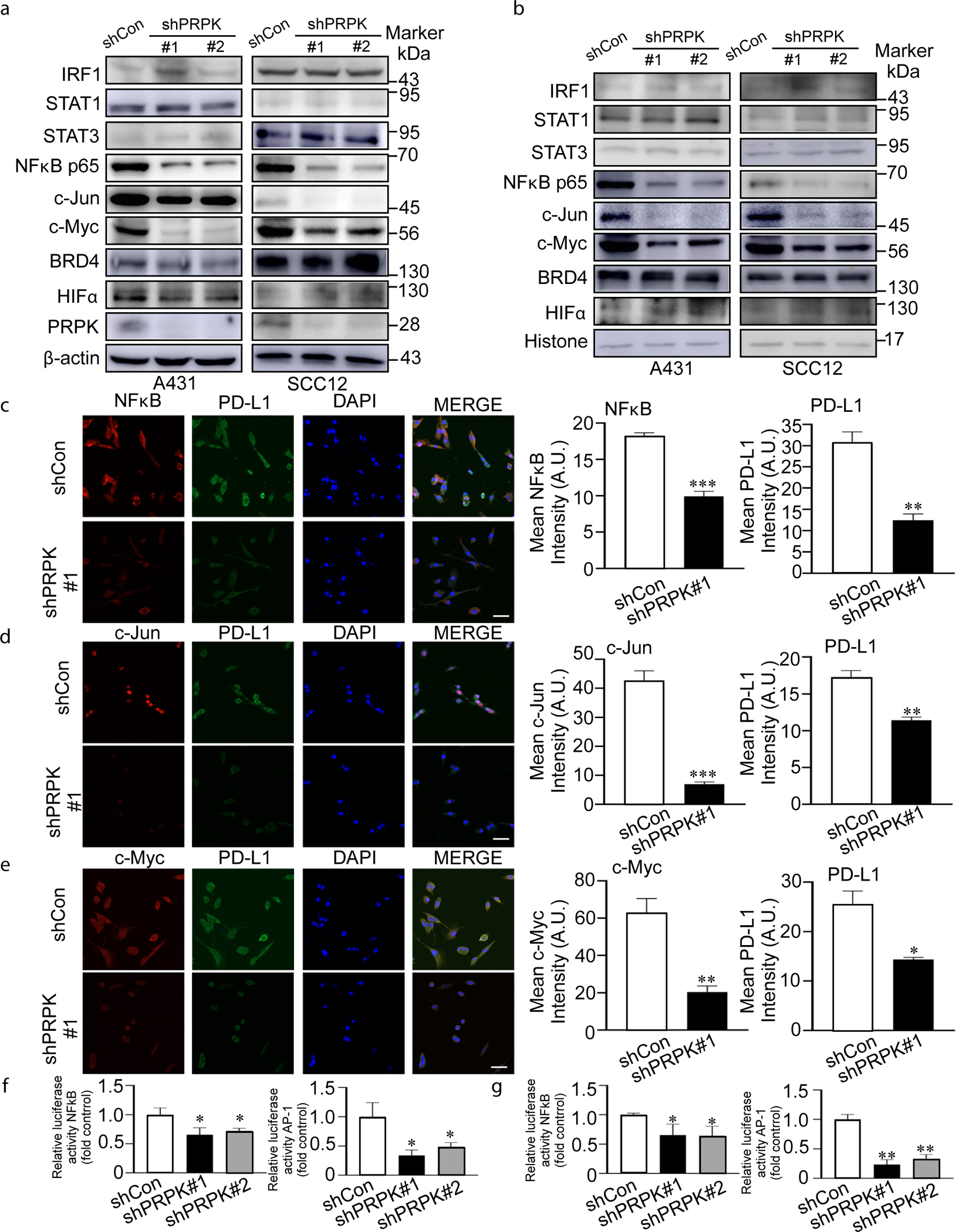
Deletion of PRPK suppresses PD-L1 expression by inhibiting transcription factor activation or expression. **(a, b)** Western blot analysis shows that the deletion of PRPK significantly reduces the (**a**) total and (**b**) nuclear levels of NF-κB, c-Jun, and c-Myc in A431 and SCC12 cells. (**c**–**e)**. Co-IF analysis demonstrates that PRPK knockdown markedly decreases the expression of NF-κB, c-Jun, c-Myc, and PD-L1. Representative images are shown for **(c)** NF-κB, **(d)** c-Jun, and **(e)** c-Myc. PD-L1 is visualized in green, transcription factors are in red, and nuclei are counterstained with DAPI (blue). Bars = 10 μm. (**f, g)** Luciferase reporter assays reveal that PRPK knockdown significantly inhibits the activation of NF-κB and AP-1 in both **(f)** A431 and **(g)** SCC12 cells. Data are presented as mean values ± SD, with statistical significance indicated (*P < .05, **P < .01, and ***P < .001; 1-way ANOVA). AP-1, activator protein 1; A.U., arbitrary unit; IF, immunofluorescence; shCon, control-targeted short hairpin RNA; shPRPK, PRPK-targeted short hairpin RNA; STAT, signal transducer and activator of transcription.

Previous studies have demonstrated that IRF1, signal transducer and activator of transcription 1, signal transducer and activator of transcription 3, NF-κB, c-Jun, c-Myc, BRD4, HIFα, and activator protein 1 are major transcription factors that can directly bind with the promoter of PD-L1 to regulate its expression (Atsaves et al, 2019; Green et al, 2012; Zerdes et al, 2018). Our results demonstrate that deletion of PRPK significantly suppresses the expression levels of NF-κB, c-Jun, and c-Myc at both total (Figure 4a) and nuclear (Figure 4b) levels, as determined by western blot analysis. In addition, co-IF assays were performed to assess the impact of PRPK on the expression of transcription factors (NF-κB, c-Jun, and c-Myc) and PD-L1. Knockdown of PRPK markedly reduces the expression levels of these transcription factors as well as PD-L1 (Figure 4c–e). Furthermore, luciferase assays revealed that knockdown of PRPK significantly inhibits the activation of NF-κB and activator protein 1 in A431 cells (Figure 4f) and SCC12 cells (Figure 4g). These findings suggest that PRPK plays a critical role in modulating transcription factor expression or activity and PD-L1 expression, which are essential for tumor progression and immune evasion.

### PRPK deletion enhances CD8+ T-cell infiltration but does not affect CD4+ T cells in tumors

To assess the effect of PRPK deletion on immune cell populations in the TME, we performed flow cytometry analysis on tumor tissues from SSL-exposed mice. Single-cell suspensions were prepared, and gating strategies were applied to include all events, live and dead cells, and immune markers (CD45, CD3, CD4, and CD8).

**Figure 5.**
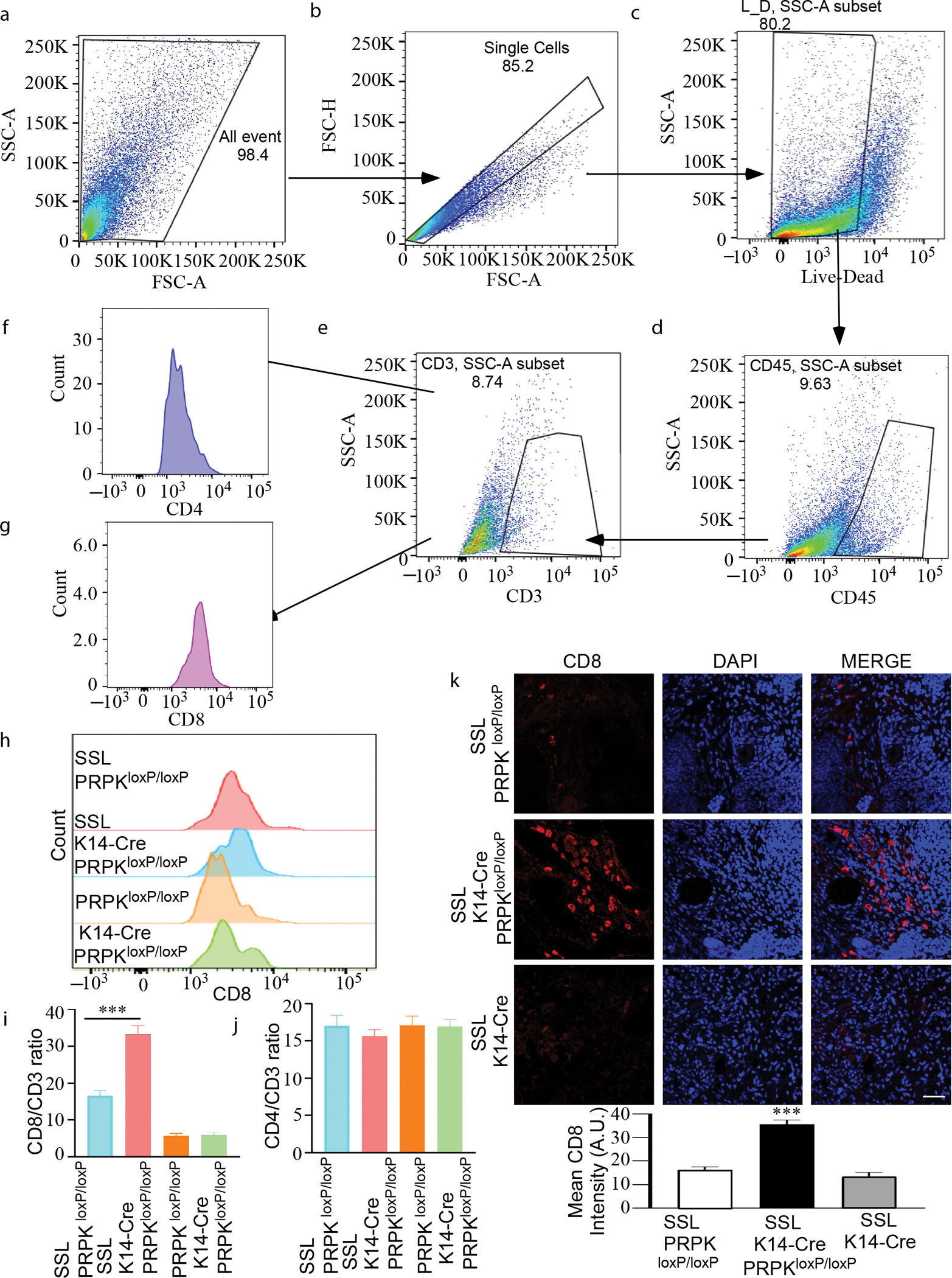
PRPK deletion enhances CD8+ T-cell infiltration in tumors. Flow cytometry analysis of immune cell populations in tumor tissues from SSL-exposed mice demonstrates the impact of PRPK deletion on the tumor microenvironment. (**a)** Total events were gated to exclude debris and doublets. (**b)** Live and dead cells were discriminated against using a viability dye. (**c)** CD45+ immune cells were gated from the live cell population. (**d)** Within the CD45+ population, CD3+ T cells were identified. (**e**–**g)** CD3+ T cells were further analyzed to separate CD4+ helper T cells and CD8+ cytotoxic T cells. (**h, i)** A significant increase in the proportion of CD8+ T cells within the CD3 immune cell population was observed in tumors from epidermis-specific PRPK-knockout mice (K14-Cre PRPK^loxP/loxP^) compared with those from control group (PRPK^loxP/loxP^). This increase was consistent across the total event population and live cell subset, indicating enhanced infiltration of CD8+ T cells into the tumor microenvironment. (**j)** No significant difference was observed in the proportion of CD4+ T cells between PRPK-knockout and control tumors. (**k)** IF analysis demonstrated the significant increase in CD8 T-cell population in PRPK-knockout tumors compared with that in control groups. Bar = 50 μm. Intensity was evaluated using the ZEISS ZEN 3.7 program. The asterisks indicate a significant (***P < .001) CD8+ increase in K14-Cre PRPK^loxP/loxP^ compared with that in the control groups. A.U., arbitrary unit; FSC-A, forward scatter area; IF, immunofluorescence; SSC-A, side scatter area; SSL, solar simulated light.

Initially, total events were gated to exclude debris and doublets (Figure 5a), followed by live/dead cell discrimination using a viability dye (Figure 5b). From the live cell population, CD45+ cells were gated to identify immune cells (Figure 5c). Within the CD45+ population, CD3+ T cells (Figure 5d and e) were further analyzed to separate CD4+ helper T cells and CD8+ cytotoxic T cells (Figure 5f and g). Our results showed a significant increase in the proportion of CD8+ T cells within the CD3+ or CD45+ immune cell population in tumors from epidermis-specific PRPK knockout mice (K14-Cre PRPK^loxP/loxP^) compared with those from control groups (PRPK^loxP/loxP^ and K14-Cre SKH1 mice). This increase was also reflected in the total event population and live cell subset, indicating enhanced infiltration of CD8+ T cells into the TME (Figure 5h and i and Supplementary Figure S4). In contrast, no significant difference was observed in the proportion of CD4+ T cells between PRPK-knockout and control tumors (Figure 5j and Supplementary Figure S4). There were no significant differences in the proportions of CD8^+^ and CD4^+^ cells in the lymph nodes between the groups (Supplementary Figure S5). In addition, the population of CD8+ T cells was significantly increased in PRPK-knockout tumors compared with that in the control groups, as demonstrated by IF analysis (Figure 5k). No difference was observed between the groups without SSL treatment (Supplementary Figure S6). These findings suggest that PRPK deletion specifically enhances CD8+ T-cell infiltration into the tumor while leaving the CD4+ T-cell population unaffected. The observed increase in CD8+ T cells, critical for antitumor immunity, highlights PRPK’s role in shaping the tumor immune microenvironment and provides a potential target for immunotherapeutic strategies.

### Modulation of the TME through the reduction of IL-6, MIP-2, and VEGF levels in PRPK-deficient tumors

**Figure 6.**
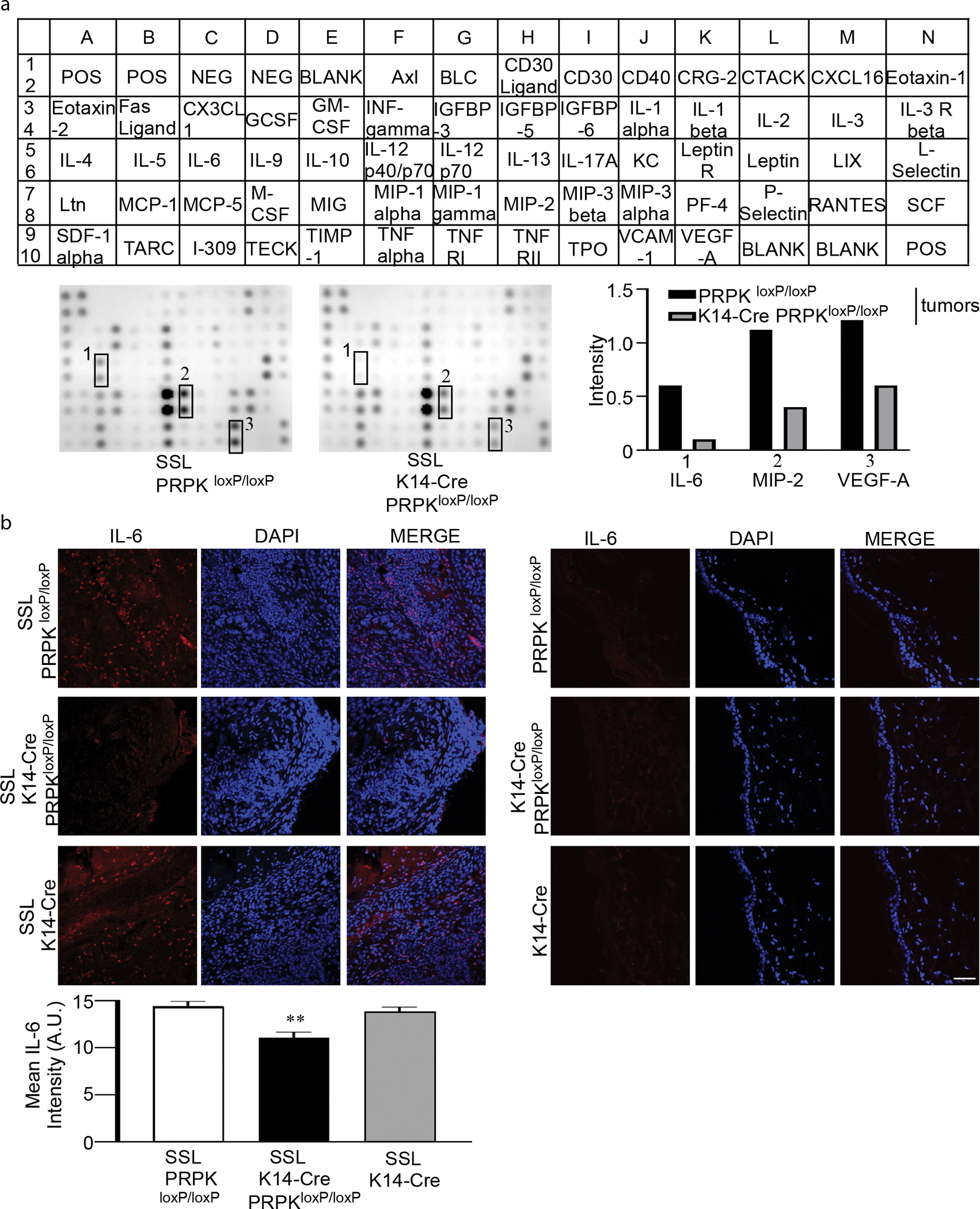
Modulation of the tumor microenvironment through reduction of IL-6, MIP-2, and VEGF levels in PRPK-deficient tumors. (**a)** Cytokine array analysis comparing K14-Cre PRPK^loxP/loxP^ KO tumors with control PRPK^loxP/loxP^ tumors shows that PRPK deletion significantly reduces the expression of proinflammatory and proangiogenic cytokines IL-6, MIP-2, and VEGF in the tumor microenvironment. **(b)** IF analysis demonstrates a marked decrease in IL-6 expression in PRPK-KO tumors compared with that in control tumors, consistent with the cytokine array results. Bar = 50 μm. Intensity was evaluated using the ZEISS ZEN 3.7 program. The asterisks indicate a significant (**P < .01) IL-6 compared with control group. A.U., arbitrary unit; IF, immunofluorescence; KO, knockout.

To investigate the impact of PRPK deletion on cytokine expression within the TME, we performed a cytokine array comparing K14-Cre PRPK^loxP/loxP^ knockout tumors with control PRPK^loxP/loxP^ tumors (Supplementary Figure S7). The results revealed that PRPK knockout markedly suppresses the expression of key proinflammatory and proangiogenic cytokines, including IL-6, MIP-2, and VEGF, compared with the control tumors (Figure 6a). To validate these findings, we assessed the expression level of IL-6 using IF. Consistent with the cytokine array results, IL-6 expression was significantly reduced in PRPK-knockout tumors compared with that in the controls (Figure 6b). These findings suggest that PRPK is pivotal in driving the proinflammatory and proangiogenic environment within the TME.

## DISCUSSION

This study highlights the critical role of PRPK in promoting solar UV–induced NMSC and contributes to a better understanding of its mechanisms and therapeutic relevance. By utilizing a CRISPR/Cas9-mediated epidermal-specific knockout mouse model and in vitro cell-based studies, we demonstrate that PRPK deficiency leads to significant suppression of tumor growth and reprogramming of the TME to favor antitumor immunity.

PRPK has been implicated in the progression of various cancers, including colon cancer, multiple myeloma, and hepatocellular carcinoma (Dwira et al, 2024; Hideshima et al, 2017; Zykova et al, 2018, 2017). Our previous research demonstrated that targeting PRPK with an inhibitor effectively suppresses SSL-induced photocarcinogenesis in mouse models (Roh et al, 2023). However, the mechanistic role of PRPK in NMSC has remained underexplored. Our findings identified PRPK as a modulator of several oncogenic processes, such as cell proliferation, by mediating transcription factors, thus revealing its impact on immune evasion and inflammation. Importantly, PRPK appears to act as a molecular nexus that integrates proliferative and immunosuppressive signaling pathways. This expands its significance beyond a tumor-intrinsic regulator to a broader orchestrator of the TME, emphasizing its potential as a therapeutic target.

The downregulation of PD-L1 observed in PRPK-deficient tumors aligns with broader findings in cancer immunology, where immune checkpoint molecules have been shown to dampen antitumor immunity (Dermani et al, 2019; Topalian et al, 2012; Wang and Wu, 2020). Our coauthors found that PD-L1 expression in keratinocytes and cSCC cells has been linked to immune suppression after solar UV exposure (Dickinson et al, 2024, 2021; García-Díez et al, 2018). Targeting PD-L1 demonstrates antitumor activity for cSCC in clinical application (Maubec et al, 2019; Munhoz et al, 2022). Our results demonstrate that deletion of PRPK can suppress PD-L1 expression levels by inhibiting the expression or activation of c-Myc, c-Jun, NF-κB, and activator protein 1. These findings show that targeting transcriptional regulators of PD-L1 can potentiate immune responses against tumors, particularly in solar UV exposure–induced tumors. CD8+ T cells are cytotoxic lymphocytes essential for antitumor immunity (Farhood et al, 2019; Raskov et al, 2021). The interaction between PD-L1 on tumor cells (or other cells in the TME) and PD-1 on CD8+ T cells suppresses CD8+ T-cell activation, proliferation, cytotoxicity, and T-cell exhaustion. Therapeutic targeting of the PD-L1/PD-1 axis using immune checkpoint inhibitors can restore CD8+ T-cell function, enhance infiltration into the TME, and reinvigorate antitumor responses (Jiang et al, 2019; Juneja et al, 2017). The enhanced infiltration of CD8+ T cells into the TME by deletion of PRPK observed in this study is particularly significant. CD8+ T cells are key effectors in tumor immunity, capable of directly killing tumor cells and shaping the immune landscape. By promoting CD8+ T-cell recruitment and reducing immunosuppressive cytokines such as IL-6 (Aliyu et al, 2022; Tilg et al, 1997), PRPK deletion effectively converts an immunosuppressive environment into an immunologically active one. In addition, to evaluate the effect of PRPK on myeloid populations, including monocytes (Ly6-C), neutrophils (Ly6-G), and dendritic cells (CD11c), our results show that PRPK deletion significantly reduced the infiltration of Ly6-C^+^ monocytes in tumors, whereas there were no significant changes observed in Ly6-G^+^ neutrophils or CD11c^+^ dendritic cells compared with that in control groups by IF staining (Supplementary Figures S8–10). Ly6C^+^ monocytes are often associated with an immunosuppressive tumor environment and can inhibit CD8^+^ T-cell activity through cytokine secretion (Jung et al, 2017). Their reduction upon PRPK deletion may help alleviate this suppressive barrier. This likely contributes to the enhanced CD8^+^ T-cell infiltration observed in PRPK-deficient tumors, suggesting a dual role for PRPK in modulating both myeloid and lymphoid compartments to promote tumor immune evasion. This reprogramming effect demonstrates the function of PRPK, which relies on sufficient immune infiltration to achieve optimal therapeutic outcomes.

In addition to immune modulation, PRPK’s suppression of proinflammatory and proangiogenic factors such as IL-6, MIP-2, and VEGF sheds light on its broader role in TME remodeling. Chronic inflammation and angiogenesis are well-established drivers of skin carcinogenesis, contributing to both tumor growth and metastasis (Hibino et al, 2021). IL-6, MIP-2, and VEGF play significant roles in shaping the TME and influencing CD8+ T-cell infiltration. IL-6 fosters an immunosuppressive TME by enhancing suppressor cells and impairing CD8+ T-cell activity (Ai et al, 2020; Tilg et al, 1997). MIP-2 (CXCL2) modulates recruitment of neutrophils and inflammatory responses, which can create a protumorigenic microenvironment (Powell and Huttenlocher, 2016). VEGF drives angiogenesis while inhibiting CD8+ T-cell infiltration by disrupting vascular integrity and supporting immunosuppressive mechanisms (Lamplugh and Fan, 2021; Zheng et al, 2022). These factors collectively promote immune evasion, tumor growth, and progression, highlighting their importance in TME-mediated regulation of antitumor immunity. The reduction in these factors suggests that PRPK inhibition disrupts the signaling networks that sustain tumor progression, offering a dual benefit of targeting tumor-intrinsic and extrinsic pathways.

**Figure 7.**
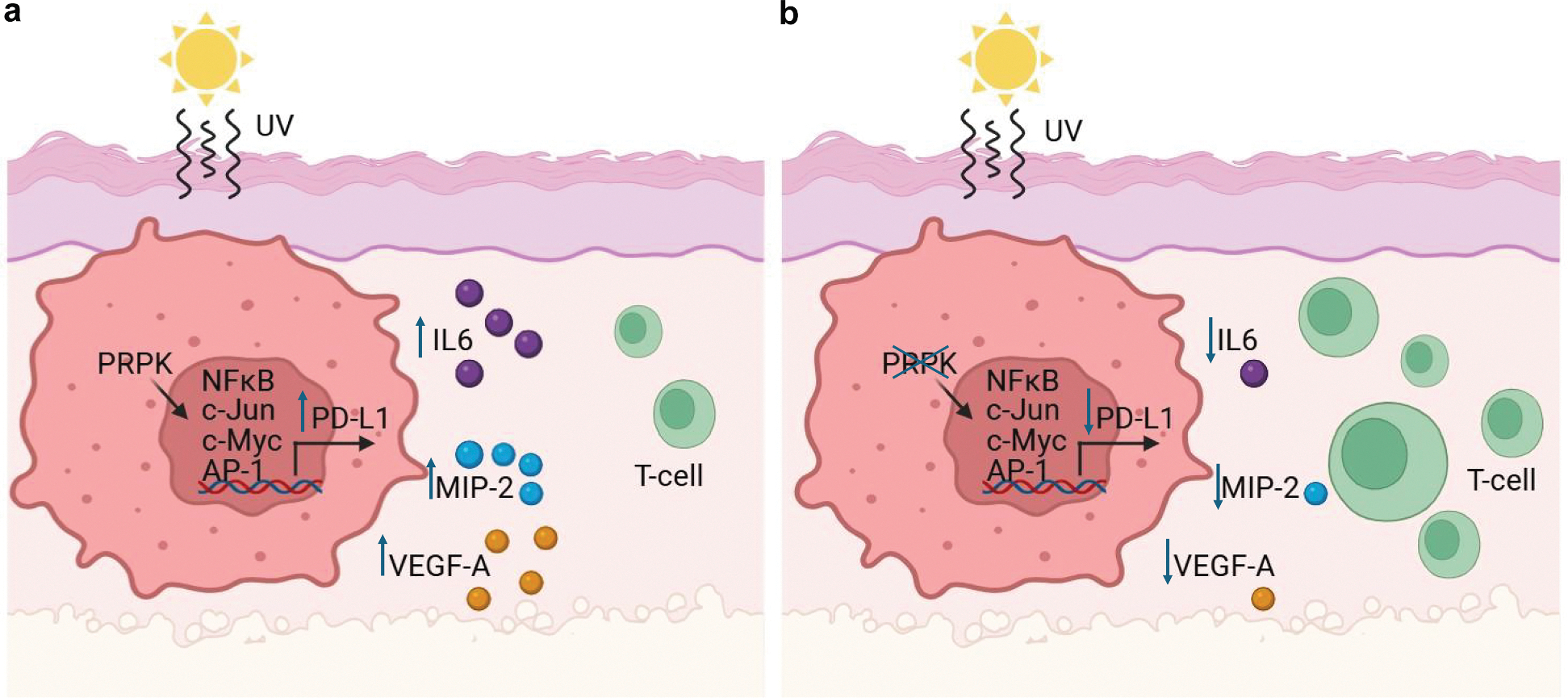
PRPK mediates solar UV–induced photocarcinogenesis by mediating PD-L1 expression and enhancing CD8+ T-cell infiltration. **(a)** This study illustrates the pivotal role of PRPK in promoting solar UV–induced photocarcinogenesis by regulating immune and inflammatory pathways within the tumor microenvironment. PRPK drives PD-L1 expression through the activation of transcription factors NF-κB, c-Jun, c-Myc, and AP-1, facilitating an immunosuppressive environment. (**b)** Deletion of PRPK disrupts this pathway, leading to enhanced CD8+ T-cell infiltration and a significant reduction in proinflammatory and proangiogenic mediators, including IL-6, MIP-2, and VEGF. These findings highlight PRPK as a critical regulator of tumor immune evasion and a potential therapeutic target for improving antitumor immunity. AP-1, activator protein 1.

In summary, this study underscores the pivotal role of PRPK in solar UV–induced NMSC, revealing its dual functions in tumor progression and immune modulation. Our ongoing studies investigating the expression of lead biomarkers in matched samples from sun-protected skin to cSCC will further reveal the time in the skin carcinogenesis at which PRPK blockade will be most effective in modulating the expression of PD-L1. Utilizing an epidermal-specific knockout model and in vitro studies, we demonstrated that PRPK deficiency suppresses tumor growth and reprograms the TME to enhance antitumor immunity. Mechanistically, PRPK deletion inhibits key transcription factors such c-Myc, c-Jun, NF-κB, and activating protor 1, reducing PD-L1 expression and facilitating CD8+ T-cell infiltration. These findings highlight PRPK as a critical mediator of immune evasion and chronic inflammation, linking it to proinflammatory and proangiogenic factors such as IL-6, MIP-2, and VEGF (Figure 7). By disrupting these pathways, PRPK inhibition not only halts tumor progression but also fosters an immunologically active TME, showcasing its potential as a preventive or therapeutic target for UV-induced skin cancers and beyond.

## MATERIALS AND METHODS

### Cell culture and transfection

The HaCaT human keratinocyte cell line and human embryonic kidney 293T cells were purchased from ATCC, whereas the human cSCC SCC12 cell line and the A431 epidermoid carcinoma cell line were purchased from Thermo Fisher Scientific. For experiments, each vial was thawed and maintained for no more than 10 passages. HaCaT and A431 cells were cultured in DMEM supplemented with 10% fetal bovine serum and 1% antibiotics, whereas SCC12 cells were maintained in a DMEM/Ham’s F-12 50/50 mix medium, also supplemented with 10% fetal bovine serum and 1% antibiotics. The methods to establish PRPK-knockdown cells were described previously (Roh et al, 2018), and details are shown in Supplementary Materials and Methods.

### Cell cycle analysis

Cell cycle distribution was determined by PI staining and flow cytometry. Briefly, A431 and SCC12 cells were harvested 48 hours after lentiviral transduction (control-targeted short hairpin RNA or PRPK-targeted short hairpin RNA), washed with PBS, and fixed in 70% ethanol at −20 °C overnight. Cells were then incubated with RNase A (100 μg/ml) and stained with PI (50 μg/ml) in PBS for 30 minutes at room temperature in the dark. Flow cytometry was performed using a BD LSRFortessa (BD Biosciences), and the percentages of cells in G1, S, and G2/M phases were quantified using FlowJo software.

### Apoptosis assay

Apoptosis was measured using Annexin V-FITC/PI dual staining followed by flow cytometry. A431 and SCC12 cells were transduced with control or PRPK-targeted short hairpin RNA, harvested 72 hours later, and stained using the Annexin V-FITC Apoptosis Detection Kit (BD Biosciences) according to the manufacturer’s instructions. Briefly, cells were washed in cold PBS, resuspended in binding buffer, and incubated with Annexin V-FITC and PI for 15 minutes at room temperature in the dark. Stained cells were analyzed using a BD LSRFortessa flow cytometer, and data were processed using FlowJo software. Early (Annexin V^+^/PI^−^) and late (Annexin V^+^/PI^+^) apoptotic cells were quantified, and total apoptosis was calculated.

### Three-dimensional spheroid cell culture

The Vitrogel Hydrogel Matrix (VHM01) from The Well Bioscience was used for 3-dimensional cell culture according to the manufacturer’s instructions. A431 and SCC12 cells were suspended in culture media, and 1 ml of Vitrogel was mixed with 500 μl of the cell suspension. The hydrogel-cell mixture (300 μl) was dispensed into a 24-well plate at a density of 1 × 10^5^ cells per well, and the gel was allowed to set at room temperature for 15 minutes. After gelation, 300 μl of medium was gently added to cover the hydrogel. The cultures were maintained at 37 °C, with medium changes every 48 hours. After 7 days, cells were harvested for IF analysis.

### Western blot analysis

Western blotting was conducted following established protocols described previously (Wang et al, 2020). Nuclear proteins were extracted using the Nuclear Extraction Kit (Cayman Chemical, item number 10009277) according to the manufacturer’s instructions, and the extracted proteins were prepared for western blot analysis. Primary antibodies were diluted 1:1000 and incubated overnight at 4 °C, followed by incubation with a horseradish peroxidase–conjugated secondary antibody diluted 1:5000. Protein bands were visualized using a chemiluminescent substrate (GE Healthcare Biosciences, Piscataway, NJ).

### Luciferase assay

A431 or SCC12 cells (control-targeted short hairpin RNA and PRPK-targeted short hairpin RNA, 8 × 10^4^ cells per well) were seeded in a 24-well plate and cultured for 24 hours. The cells were then transfected with NF-κB or activator protein 1 luciferase reporter plasmids, along with a β-galactosidase plasmid as an internal control, using iMFectin Poly DNA Transfection Reagent (GenDEPOT) according to the manufacturer’s instructions. After 24 hours, luciferase and β-galactosidase activity levels were measured using the Luminoskan Ascent and Multiskan MCC systems (Labsystems Diagnostics). Luciferase activity was normalized to β-galactosidase activity to ensure reliable and accurate results.

### SSL-induced cSCC mouse models

All animals were maintained in compliance with guidelines approved by the University of Minnesota Institutional Animal Care and Use Committee (protocol identification 2205–40061A). They were housed in climate-controlled conditions with a 12-hour light/dark cycle.

Skin carcinogenesis experiments were conducted on mice aged 6–8 weeks. Carcinogenesis was induced using a SSL irradiation system that has been described previously (Gao et al, 2017; Roh et al, 2018). The solar UV source (Q-Lab) emitted light in the 295–365 nm range, with peak intensity at 340 nm. Mice were exposed to gradually increasing doses of SSL over time. In the first week, they received 36 kJ/m^2^ of UVA and 1.8 kJ/m^2^ of UVB. The dose was increased by 10% each week, reaching 60 kJ/m^2^ UVA and 2.9 kJ/m^2^ UVB by week 6. This dose was then maintained consistently from week 6 to week 16. Group sets were organized as follows: group 1 comprised PRPK^loxP/loxP^ (8 male and 8 female) with SSL, group 2 comprised K14-Cre PRPK^loxP/loxP^ (8 male and 8 female) with SSL, group 3 comprised K14-Cre (7 male and 7 female) with SSL, group 4 comprised PRPK^loxP/loxP^ (5 male and 5 female) without SSL, group 5 comprised K14-Cre PRPK^loxP/loxP^ (5 male and 4 female) without SSL, and group 6 comprised K14- (5 males and 4 females) without SSL (Figure 1e). Mice were weighed, and tumors were measured weekly until week 29, when the total tumor volume reached 2 cm^3^ or when ulceration occurred. At that point, the mice were killed. Portions of each tumor sample were immediately fixed in 10% formalin for IF analysis, whereas the other portions were used for cytokine array and flow cytometry. The remaining tissues were frozen for further analysis.

### Mouse cytokine antibody array

RayBio Mouse Cytokine Antibody Arrays C3 (RayBiotech) analyses were conducted following the manufacturer’s instructions and using tissue lysates pooled (8 males and 8 females for each group) from multiple animal samples. Protein concentrations in the lysates were determined with a Protein Assay Kit (Bio-Rad Laboratories), ensuring 500 μg of protein per sample. Lysates were diluted in blocking buffer to a final concentration of 5 μg/μl. The details are provided in Supplementary Materials and Methods.

### Flow cytometry

For each sample (6 males and 6 females randomly selected from each group), 3 million cells were plated per well in a 96-well plate. The cells were centrifuged at 400*g* for 5 minutes at room temperature, and the supernatant fraction was discarded. A fixable viability stain (1 μl in 1 ml PBS) was prepared, with 50 μl was added to each sample for live/dead staining. The samples were incubated in the dark at room temperature for 10–15 minutes. After staining, 50–100 μl of FACS buffer was added to each well, followed by centrifugation at 400*g* for 5 minutes, and the supernatant fraction was discarded. Next, 50 μl of antibody solution was added to each well, and the samples were incubated at 4 °C for 30–40 minutes. After incubation, 100 μl of FACS buffer was added, and the cells were centrifuged at 400*g* for 5 minutes at room temperature. This washing step, involving the addition of 100 μl FACS buffer followed by centrifugation, was repeated 3 times. After the final wash, the supernatant fraction was removed, and the cells were resuspended in 2.5% FACS buffer. Flow cytometric data were collected using a BD LSR Fortessa flow cytometer and analyzed with FlowJo software.

The reagents and antibodies, IF analysis of the MTT assay, 3-dimensional spheroids and 2-dimensional monolayers in A431 and SCC12 cells and mouse tissue, H&E staining, mouse PRPK conditional knockout model generation, and the cell isolation for flow cytometry are described in Supplementary Materials and Methods.

### Statistical analysis

Quantitative data are expressed as means ± SD or standard error on the basis of a minimum of 3 independent experiments or samples. Significant differences were evaluated using either a student’s *t*-test or 1-way ANOVA. A *P* < .05 was considered statistically significant.

## Supplementary Material

1

SUPPLEMENTARY MATERIAL

Supplementary material is linked to the online version of the paper at www.jidonline.org, and at 10.1016/j.jid.2025.07.021.

## Data Availability

The data that support the findings of this study are available from the corresponding author Dr. Tianshun Zhang, email: zhan4145@umn.edu upon reasonable request.

## Abbreviations:

cSCC: cutaneous squamous cell carcinoma
IF: immunofluorescence
NMSC: nonmelanoma skin cancer
PI: propidium iodide
SSL: solar simulated light
TME: tumor microenvironment
