# Supplementary material for: Deletion of p53-Related Protein Kinase Suppresses Solar UV–Induced Photocarcinogenesis by Inhibiting PD-L1 Expression and Enhancing CD8 T-Cell Infiltration
Source: J Invest Dermatol. Author manuscript; Available in PMC 2025 Dec 19. (PMC12421728; doi:10.1016/j.jid.2025.07.021)
Supplement: 1 [file NIHMS2104690-supplement-1.pdf]

Supplementary Materials and Methods

Reagents and antibodies.

For cell culture, essential materials, such as media, gentamicin, and penicillin, were purchased from Invitrogen, while MEM non-essential amino acids were purchased from Corning (NY). Fetal bovine serum (FBS) was sourced from Gemini Bio-Products (West Sacramento, CA). Laboratory reagents, including Tris, NaCl, sodium bicarbonate, hydrocortisone, glucose, transferrin, collagenase IV, deoxyribonuclease I, trypsin-EDTA solution (T4049), and SDS, were purchased from Sigma-Aldrich (St. Louis, MO) for use in molecular biology assays and buffer preparation. Antibodies used in the experiments included β-actin (sc-47778), PRPK (sc-100350), IRF1 (sc-137061), BRD4 (sc-518021), histone H3 (sc-517576), and IL-6 (sc-130326) from Santa Cruz Biotechnology; PCNA (#13110), STAT1 (#9172), STAT3 (#2523), c-Jun (#9165), c-Myc (#13987), p-p53 ser15 (#9286) and NF-κB (p65) (#8242) from Cell Signaling Technology (Danvers, MA); and PD-L1 (NBP1-43262) and HIF-1α (NB100-479) from Novus. For Western blotting, TrueBlot Ultra antibodies were used, along with secondary antibodies including anti-rabbit Ig HRP (18-8816-31) and anti-mouse Ig HRP (18-8817-31) purchased from Rockland (Rockland, ME). For immunofluorescence (IF) analysis, Ly-6G (#127601) and Ly-6C (#128001) antibodies were purchased from BioLegend (San Diego, CA). CD11c antibody (C565591) was obtained from BD Biosciences (San Jose, CA). The secondary antibodies, including anti-rat (A11006), anti-rabbit (A32740), and anti-mouse (A11005), were purchased from Invitrogen. For flow cytometry (FACS), antibodies for Live/Dead staining (565388), CD45 (564590), CD3 (558214), CD4 (553046), and CD8 (566985) were purchased from BD Bioscience (San Jose, CA). These reagents and antibodies were essential for the successful completion of the molecular and cellular experiments conducted in this study.

Cell culture and transfection

HEK293T cells were transfected with either PRPK-targeting lentiviral vectors (shPRPK) or the pLKO.1-puro non-target shRNA control plasmid (shNT), along with the packaging vectors pMD2.0G and psPAX. Transfection was conducted using iMfectin Poly DNA transfection reagent (GenDEPOT, Barker, TX) according to the manufacturer's instructions. The transfection mixture, prepared in DMEM with 10% FBS and no antibiotics, was applied to the cells for 12 hours. Afterward, the medium was replaced with fresh DMEM containing antibiotics. Viral supernatant fractions were collected 48 hours later, filtered through a 0.45-μm filter, and used to infect target cells in the presence of 8 μg/mL polybrene (Millipore). Infected cells were selected with puromycin 24 hours before being used for further experiments.

Cell Viability (MTT) Assay

Journal Pre-proof

Cells (A431 and SCC12) were seeded in 96-well plates at a density of 3×10^3 cells per well and allowed to attach overnight. Cell viability was assessed at 24, 48, 72, and 96 hours by using the MTT assay. Briefly, 20 µL of 5 mg/mL MTT reagent (Sigma-Aldrich) was added to each well and incubated at 37°C for 1 hour. Absorbance was measured at 492 nm using a microplate reader (Thermo Fisher Scientific). Data were normalized to the 24-hour time point and presented as mean ± SE from three independent experiments.

Immunofluorescence analysis of 3D spheroids and 2D monolayers in A431 and SCC12 cells

3D Spheroid Culture: For 3D cultures, the cover medium was carefully removed from the hydrogel surface, followed by 3 washes with 100 µL of PBS, each allowed to sit on the hydrogel for 1 minute before being discarded. The spheroids were fixed with 100 µL of 4% formaldehyde solution for 30 minutes at room temperature, then washed 3 more times with PBS. Permeabilization was performed using 100 µL of 0.1% Triton X-100 for 5 minutes at room temperature, followed by 3 additional PBS washes. Blocking was achieved with 3% BSA in PBS for 60 minutes. Primary antibodies against PRPK and PD-L1 were added directly to the blocking solution and incubated overnight at 4°C. After 3 PBS washes, secondary antibodies (Alexa Fluor 488 rat IgG or Alexa Fluor 594 mouse IgG conjugates) were applied for 5 hours, followed by DAPI staining for nuclear visualization (5 minutes in the dark). The samples were imaged using a fluorescence microscope.

2D Monolayer Culture: A431 or SCC12 cells (4×10^4 cells/well) stably expressing shCon or shPRPK were seeded onto 4-chamber slides. After 24 hours, cells were washed with PBS, fixed with methanol for 20 minutes, and rinsed twice with PBST (PBS containing 0.05% Tween-20). Blocking was performed using 10 mg/mL BSA in PBS for 30 minutes. Cells were then incubated overnight at 4°C with primary antibodies (NF-κB, c-Jun, or c-Myc at 1:200) alongside PD-L1 (1:200). After washing with PBST, cells were blocked with 2% goat serum in BSA/PBS for 20 minutes. Secondary antibodies (Alexa Fluor 488 rat IgG or Alexa Fluor 594 rabbit/mouse IgG conjugates) were applied for 45 minutes, followed by DAPI staining with Fluoro-gel II for 30 minutes at room temperature. The imaging was performed using a Zeiss Axioskop microscope.

Hematoxylin and Eosin (H&E) staining and Immunofluorescence analysis of mouse tissue

Paraffin-embedded skin tissue sections were deparaffinized in xylene and rehydrated through a graded ethanol series (100%, 95%, 70%) to distilled water. For histological analysis, Hematoxylin and Eosin (H&E) staining was performed. Slides were stained with Hematoxylin (GHS216, Millipore Sigma) for 3 minutes, rinsed in running tap water, and differentiated in 1% acid alcohol if needed. After bluing in running water or bluing reagent, sections were counterstained with Eosin Y (HT110132, Millipore Sigma) for 30 seconds to 1 minute. Slides were then dehydrated through graded alcohols, cleared in xylene, and cover slipped with

Journal Pre-proof

mounting medium. This staining was used to evaluate tissue morphology, inflammation, and tumor architecture. For immunofluorescence analysis, antigen retrieval was conducted by boiling slides in sodium citrate buffer (10 mmol/L, pH 6.0) for 15 minutes. Slides were blocked with 10% goat serum albumin in PBS for 1 hour at room temperature, then incubated overnight at 4°C with primary antibodies (PCNA at 1:2000; PD-L1, CD8, or IL-6 at 1:100). After washing, slides were incubated with secondary antibodies (anti-rat or anti-mouse IgG at 1:1000, Invitrogen) for 1 hour at room temperature. The TrueView Autofluorescence Quenching Kit (Vector Laboratories) was used to minimize background fluorescence, and slides were visualized to detect target proteins.

Mouse PRPK conditional knockout model generation

CRISPR guide RNAs, used for mouse PRPK gene conditional knockout were designed by using the CRISPOR (https://crispor.gi.ucsc.edu/) web tool. Two guides were selected for introducing the long single-stranded DNA (lssDNA) containing 5' and 3'loxP sites floxing Exon 2 of the mouse gene: CTGGCACCTATCAACCACTA AGG (5'lxP gRNA on the reverse strand) and GCGGAATGGAGCTAGGACGT GGG (3'gRNA2 on the forward strand), (PAM sequence in green). Synthetic single guide RNAs were made by Synthego (https://www.synthego.com/). eSpCas9 recombinant protein was ordered from Millipore (ESPCAS9PRO-250UG). lssDNA was used as a donor for the CRISPR/Cas9 homology-directed repair. The 3.3 kb lssDNA was made by using Takara's the Guide-it Long ssDNA Production System (https://www.takarabio.com/products/gene-function/gene-editing/crispr-cas9/long-ssdna-for-knockin). Ribonucleoprotein complexes (RNP) were assembled by incubating the recombinant Cas9 protein with sgRNA for 15 minutes at room temperature. Following this, the lssDNA donor was added to the mixture, followed by 10 minutes of centrifugation at 10,000 rpm. The final concentrations for microinjections were 50 ng/µL, 30 ng/µL, and 10 ng/µL, respectively. Fertilized eggs were collected from the oviducts of super-ovulated SKH-1 females. Microinjections were performed by using a continuous flow injection of the RNP/lssDNA donor mixture into the pronucleus of 1-cell zygotes. Tail-tipping of the newborn mice was conducted to purify genomic DNA for genotyping by PCR, utilizing two screening primers: a forward primer (5'-AAGGAGTCGGAGGCATGG) and a reverse primer (5'-ACTGTCTCCTGCTCTCTAACAACC), which produced a 3.7 kb band. Positive mice exhibited three bands measuring 889 bp, 1.7 kb, and 994 bp after the PCR reactions were digested with XhoI and AscI restriction enzymes adjacent to the loxP sites. Three female founders were generated, and two of them, F4 and F5, successfully transmitted the targeted allele to the F1 generation. To generate epidermal-specific PRPK knockout mice, the SKH1 hairless mice with loxP-flanked PRPK alleles (PRPKloxP/loxP) were crossed with keratin 14-Cre (K14.Cre) mice.

Mouse cytokine antibody array

Journal Pre-proof

RayBio® Mouse Cytokine Antibody Arrays C3 (RayBiotech, Inc.) analyses were conducted following the manufacturer's protocol and using tissue lysates pooled (8 male and 8 females for each group) from multiple animal samples. Protein concentrations in the lysates were determined with a Protein Assay Kit (Bio-Rad Laboratories), ensuring 500 µg of protein per sample. Lysates were diluted in blocking buffer to a final concentration of 5 µg/µL.

Array membranes were pre-incubated with blocking buffer for 30 minutes, then the buffer was removed. Each assay well received 1 mL of the diluted protein sample and incubated overnight at 4°C. Following incubation, the samples were removed, and the membranes were washed 3 times with 2 mL of Wash Buffer I (5 minutes per wash at room temperature), followed by 2 mL of Wash Buffer II under the same conditions.

Next, 1 mL of biotin-conjugated antibodies were added to each assay well, and the membranes were incubated overnight at 4°C. After another round of washing with Wash Buffers I and II, 2 mL of HRP-streptavidin solution were added to each well. The membranes were covered with adhesive film and aluminum foil to protect them from light and incubated for 2 hours at room temperature. The membranes were then washed again with Wash Buffers I and II, followed by chemiluminescent detection.

Signal densities for each spot were quantified by using ImageJ software, and data analysis was performed as outlined in the protocol.

Journal Pre-proof

Cell isolation for flow cytometry

The tumor samples were finely chopped into small pieces in 6 cm dishes and immersed in 3 mL of digestion solution containing 0.5 mg/mL collagenase and 1 mg/mL DNase prepared in RPMI-1640. The samples were then incubated for 2 hours at 37°C in a cell culture incubator. 1 mL of 0.25% Trysin-EDTA was added into each well for the last 10 minutes of incubation. The enzymatic reaction was deactivated by adding 4 mL of 4°C 2.5% FACS buffer (PBS containing 2.5% FBS). The sample was mechanically dissociated with a 12 mL syringe by pumping 8-10 times. Lymph nodes were isolated from mice and mashed through 70 µm nylon cell strainers to remove clumps by using PBS. Cells were spun down at 400 g for 8 minutes, and the supernatant fraction was removed. After resuspending the pellet, cells were used for flow cytometry. Excessive force was avoided, and the cell suspension was filtered through 100 µm nylon cell strainers. Cells were again spun down at 400 g for 8 minutes, the supernatant fraction was removed, and cells resuspended in 10 mL of 2.5% FACS buffer. The suspension was filtered again with 40 µm nylon cell strainers and cells spun down at 400 g for 8 minutes. The supernatant fraction was removed and resuspended with 300 mL of 2.5% FACS buffer.

Supplementary Figure Legends

Supplementary Figure S1. Mice identification. a. Genotyping results for the mice in Group1: PRPKloxP/loxP (8 male and 8 female) with SSL; Group 2: K14-Cre PRPKloxP/loxP (8 male and 8 female) with SSL; Group 3: K14-Cre (7 male and 7 female) with SSL; Group 4: PRPKloxP/loxP (5 male and 5 female) without SSL; Group 5: K14-Cre PRPKloxP/loxP (5 male and 4 female) without SSL; Group 6: K14- (5 male and 4 female) without SSL. **b.** The primer information for the K14-Cre and PRPK. **c.** The PCR condition for genotyping. **d.** The epidermis was collected from mouse skin using scalpels on dry ice. The expression of PRPK (SC-85846) and β-actin (SC-47778) in pooled samples (randomly selected 2 males and 2 females from each group) was analyzed by Western blot.

Journal Pre-proof

Supplementary Figure S2. Effects of PRPK on PCNA and PD-L1 expression in mice without exposure to SSL irradiation. IF analysis was conducted to evaluate the expression levels of (**a**) PCNA and (**b**) PD-L1 in skin from K14-Cre, PRPKloxP/loxP mice and control groups (PRPKloxP/loxP or K14) in the absence of SSL irradiation. The scale bar = 50 μm.

Supplementary Figure S3. Knockdown of PRPK suppresses DMBA-induced expression of PD-L1 and phosphorylation of p53 at Ser15 in HaCaT cells. shPRPK and shControl HaCaT cells were treated with DMBA for 24 hours. Knockdown of PRPK reduced the expression of PD-L1 and phosphorylated p53 (Ser15).

Supplementary Figure S4. Quantification of $CD4^+$ and $CD8^+$ T cells among $CD45^+$ immune cells. The proportions of $CD4^+$ and $CD8^+$ T cells within the $CD45^+$ immune cell population (as shown in Fig. 5) were analyzed and compared between groups.

Supplementary Figure S5. Effect of PRPK on T cell population in lymph nodes. Flow cytometry analysis of T cell populations in lymph nodes from mice. **a**. Total events were gated to exclude debris and doublets. **b.** Live and dead cells were differentiated by using viability dye. **c.** CD45+ immune cells were gated from the live cell population. **d.** Within the CD45+ population, CD3+ T cells were identified. **e, f, g.** CD3+ T cells were further analyzed to separate CD4+ helper T cells and CD8+ cytotoxic T cells. h, i. No significant difference was observed in the proportion of CD8+ or CD4+ T cells between lymph nodes from epidermis-specific PRPK knockout mice (K14-Cre PRPKloxP/loxP) compared to the control group (PRPKloxP/loxP) with or without SSL irradiation.

Supplementary Figure S6. Effects of PRPK on CD8 T cell population in mice without SSL irradiation. IF analysis demonstrated the level of CD8 T cell population in PRPK knockout tumors compared to control groups. The scale bar = 50 µm.

Supplementary Figure S7. PRPK modulates the tumor microenvironment as revealed by cytokine array analysis. Cytokine levels were compared between tumor samples from PRPK knockout mice (K14-Cre PRPKloxP/loxP) compared to control group (PRPKloxP/loxP) by using a mouse cytokine array. The immunoblotting spot intensities were quantified using ImageJ; and the expression levels of various cytokines are presented in graphical form.

Supplementary Figure S8. Effect of PRPK deletion on Ly6-C monocyte populations in mice with or without SSL irradiation exposure. IF analysis revealed the levels of Ly6-C monocyte population in PRPK knockout tumors compared to control groups. The scale bar = 50 µm. Intensity was evaluated by using the ZEISS ZEN 3.7 program. The asterisks indicate a significant (**, $p < 0.01$) Ly6-C monocyte decreased in K14-Cre PRPKloxP/loxP compared to the control groups.

Supplementary Figure S9. Effect of PRPK deletion on Ly6-G neutrophil populations in mice with or without SSL irradiation exposure. IF analysis revealed the levels of Ly6-G neutrophils population in PRPK knockout tumors compared to control groups. The scale bar = 50 µm. Intensity was evaluated by using the ZEISS ZEN 3.7 program.

Supplementary Figure S10. Effect of PRPK deletion on CD11c dendritic cells populations in mice with or without SSL irradiation exposure. IF analysis revealed the levels of CD11c dendritic cells population in PRPK knockout tumors compared to control groups. The scale bar = 50 µm. Intensity was evaluated by using the ZEISS ZEN 3.7 program.

Journal Pre-proof

Supplementary Figure 1

a

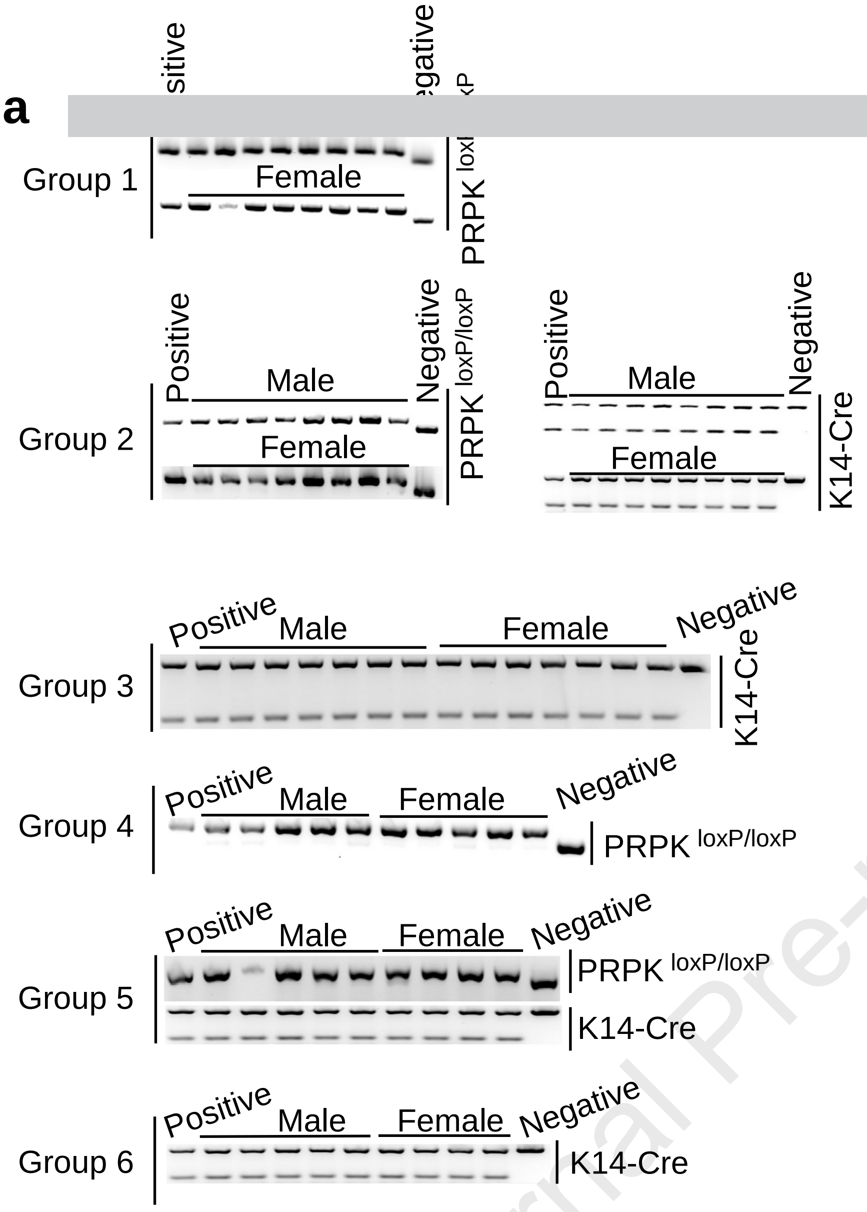

Step	Temp °C	Time	
1	94	2:00	
2	94	0:20	
3	65	0:15	-0.5°C per cycle
4	68	0:10	
5	Goto Step2, 10X		
6	94	0:15	
7	60	0:15	
8	72	0:10	
9	Goto Step6, 28X		
10	72	2:00	
11	10	Keep	

b

primer	Sequence (5' to 3' direction)
Cre F	GCGGTCTGGCAGTAAAAACTATC
Cre R	GTGAAACAGCATTGCTGTCACTT
Internal Positive Control F	CTAGGCCACAGAATTGAAAGATCT
Internal Positive Control R	GTAGGTGGAAATTCTAGCATCATCC
PRPK F	GAGTTCAGGTGATTAGGCTTGG
PRPK R	AGGCACTAATGTAGCCTCTGTAACC

d

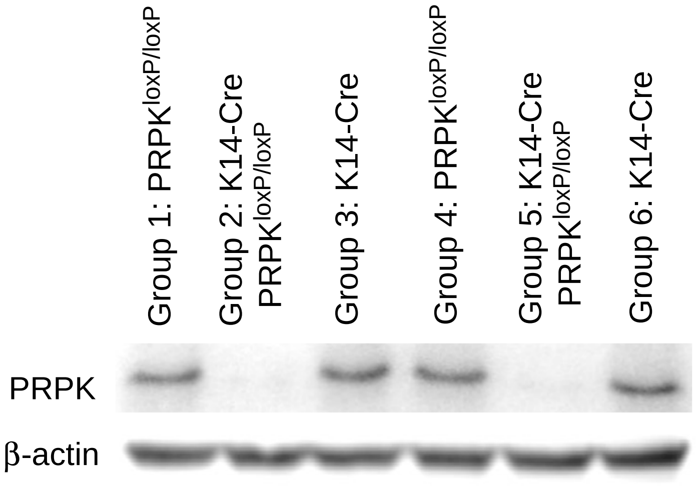

Journal Pre-proof

Supplementary Figure 2

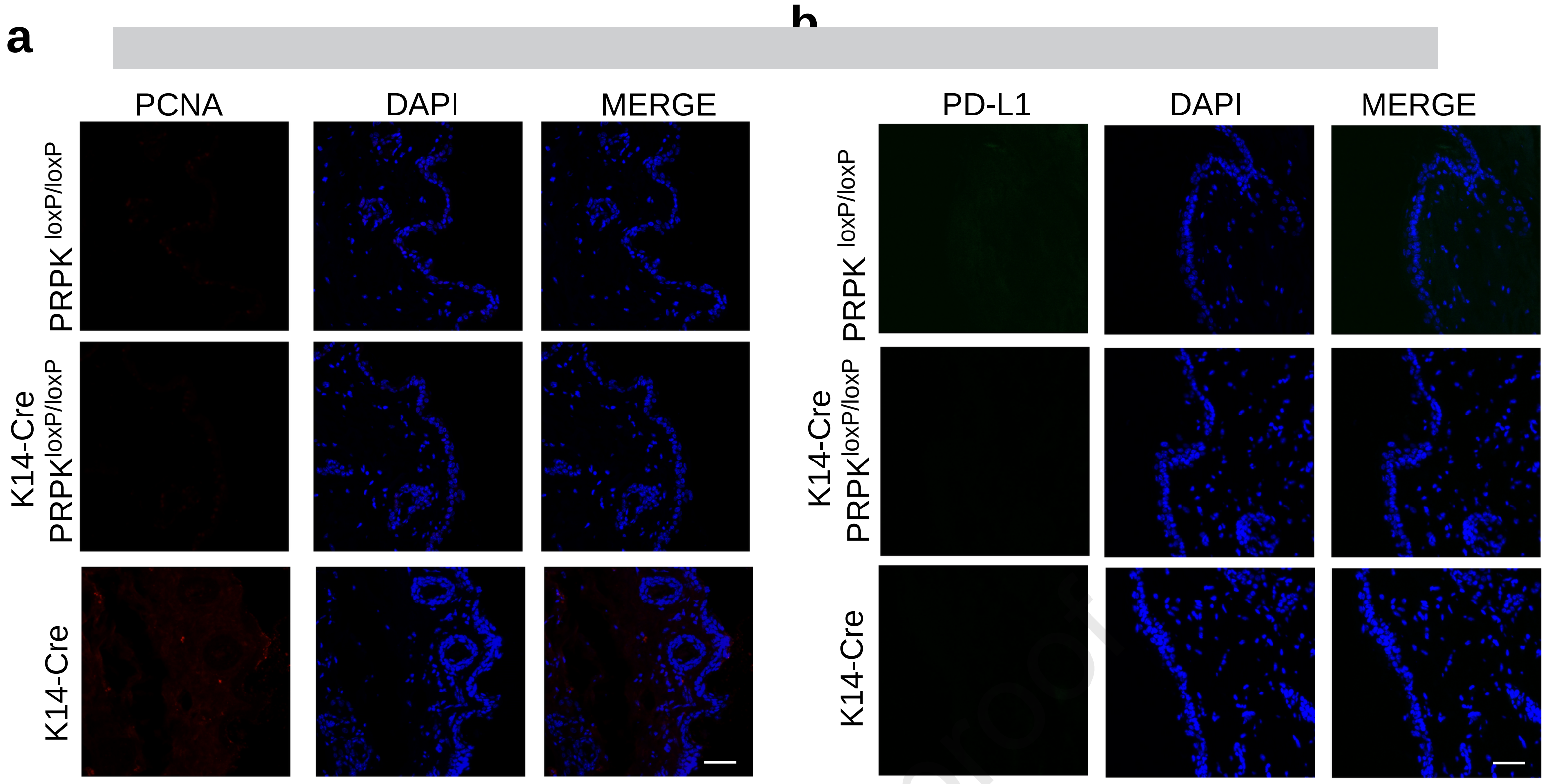

Journal Pre-proof

Supplementary Figure 3

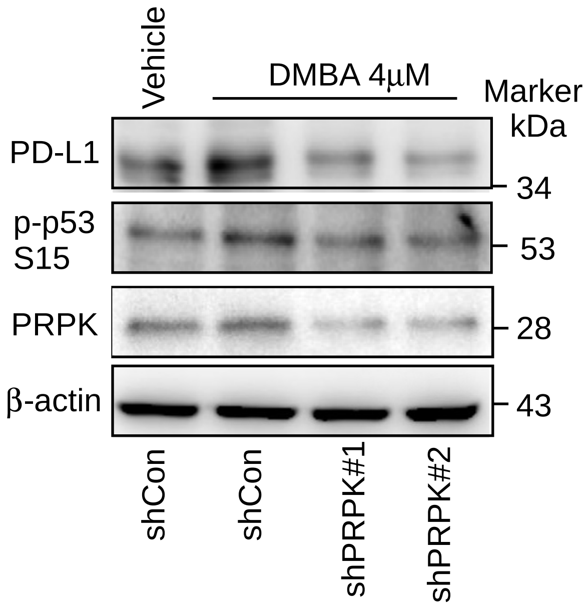

Journal Pre-proof

Supplementary Figure 4

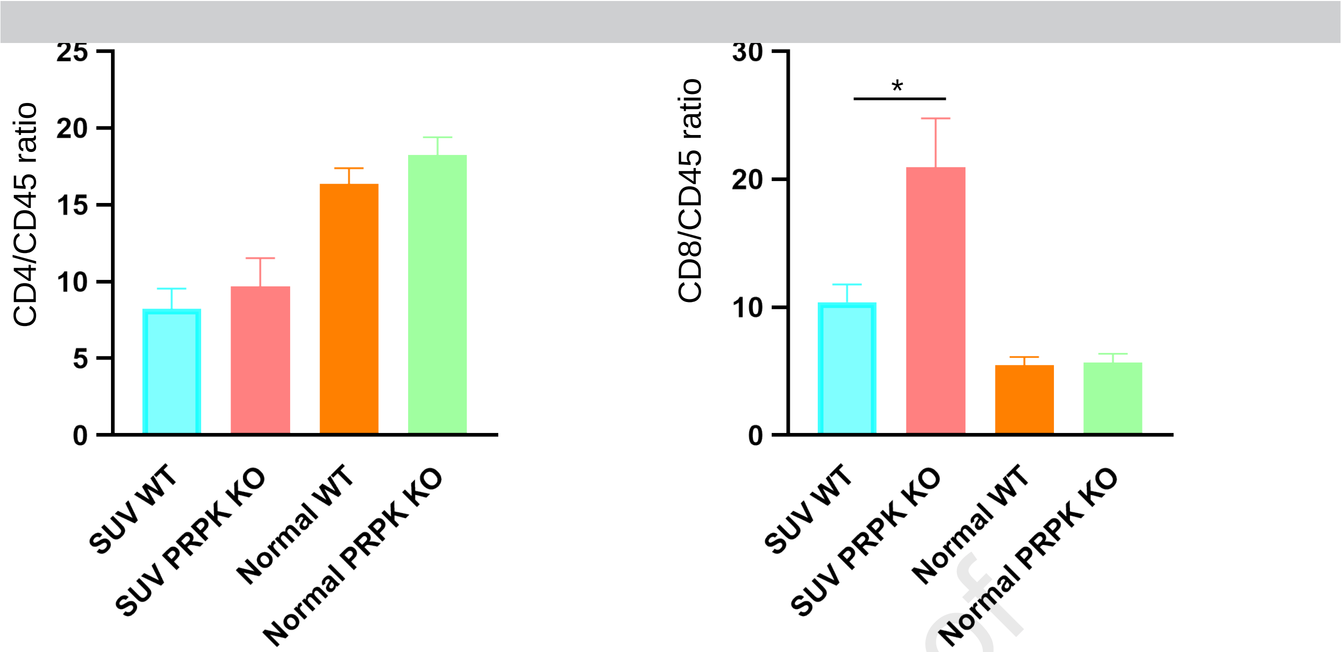

Journal Pre-proof

Supplementary Figure 5

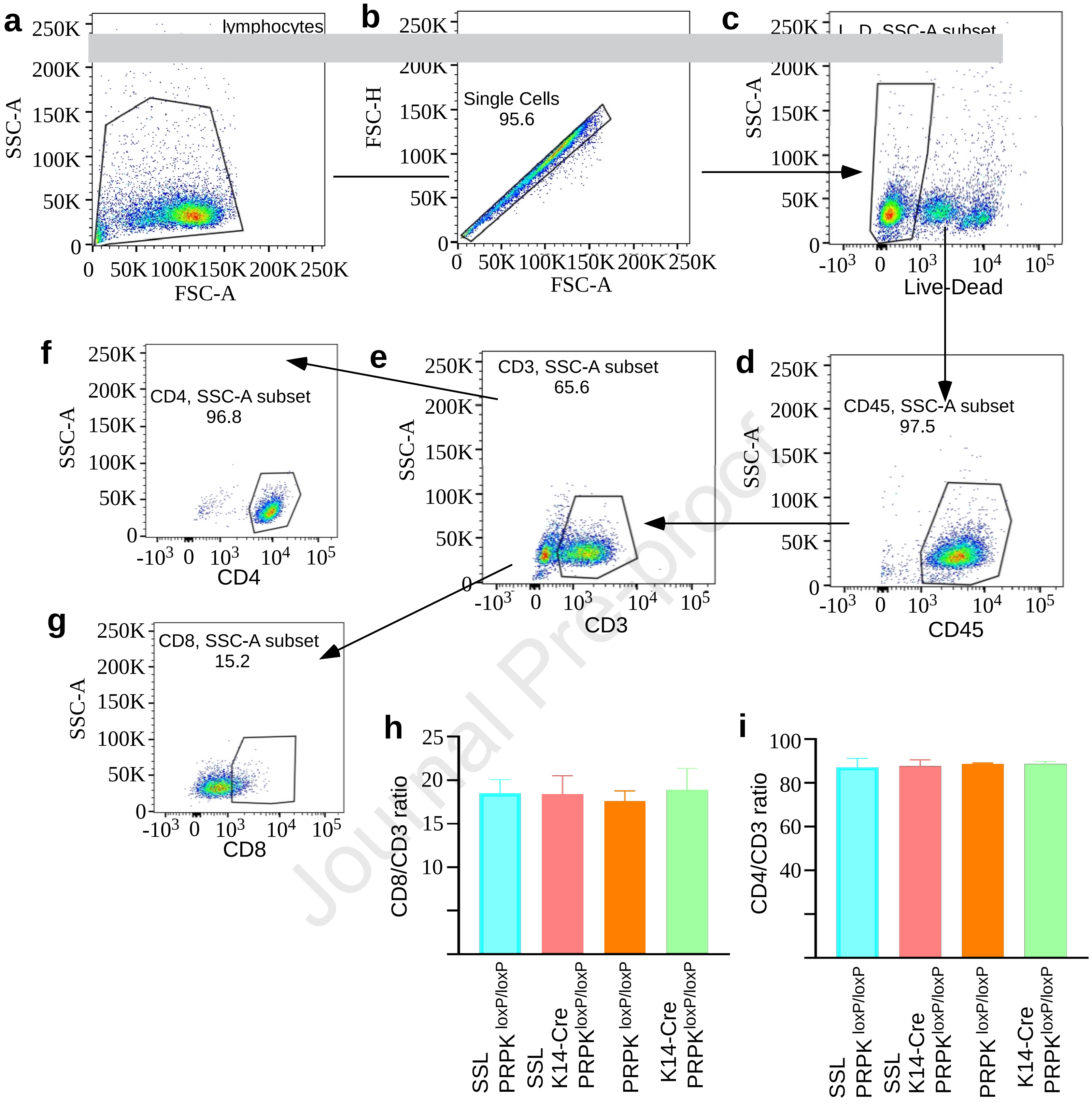

Journal Pre-proof

Supplementary Figure 6

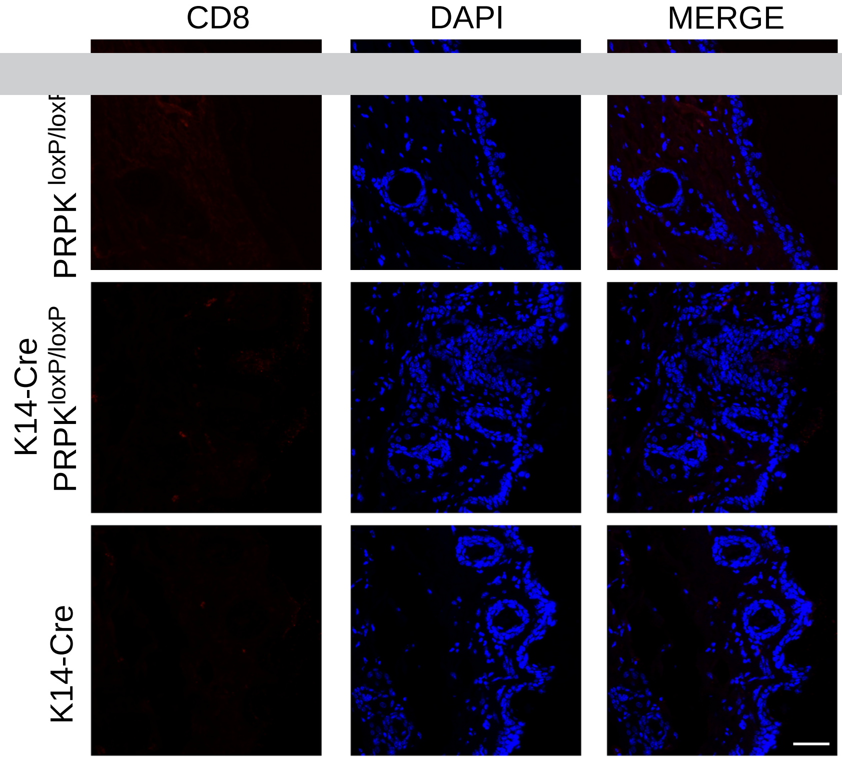

Journal Pre-proof

Supplementary Figure 7

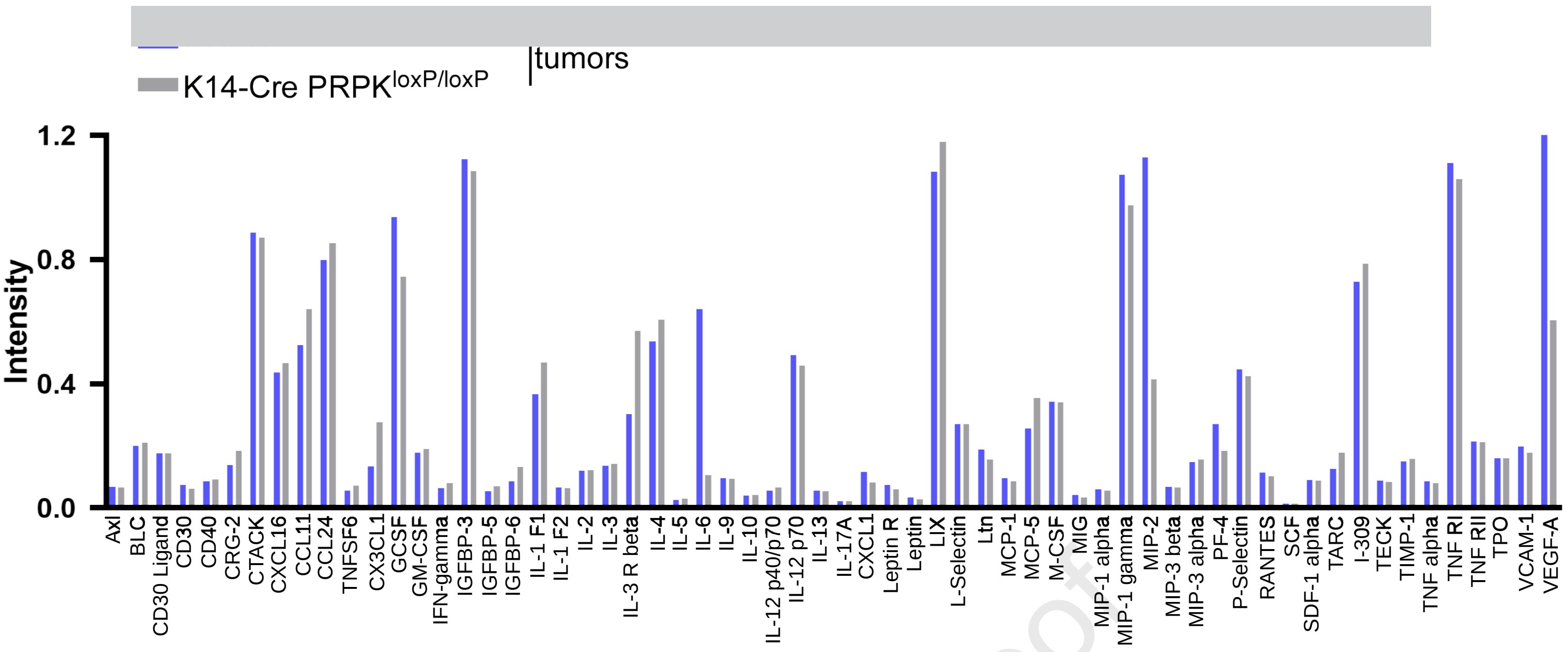

Journal Pre-proof

Supplementary Figure 8

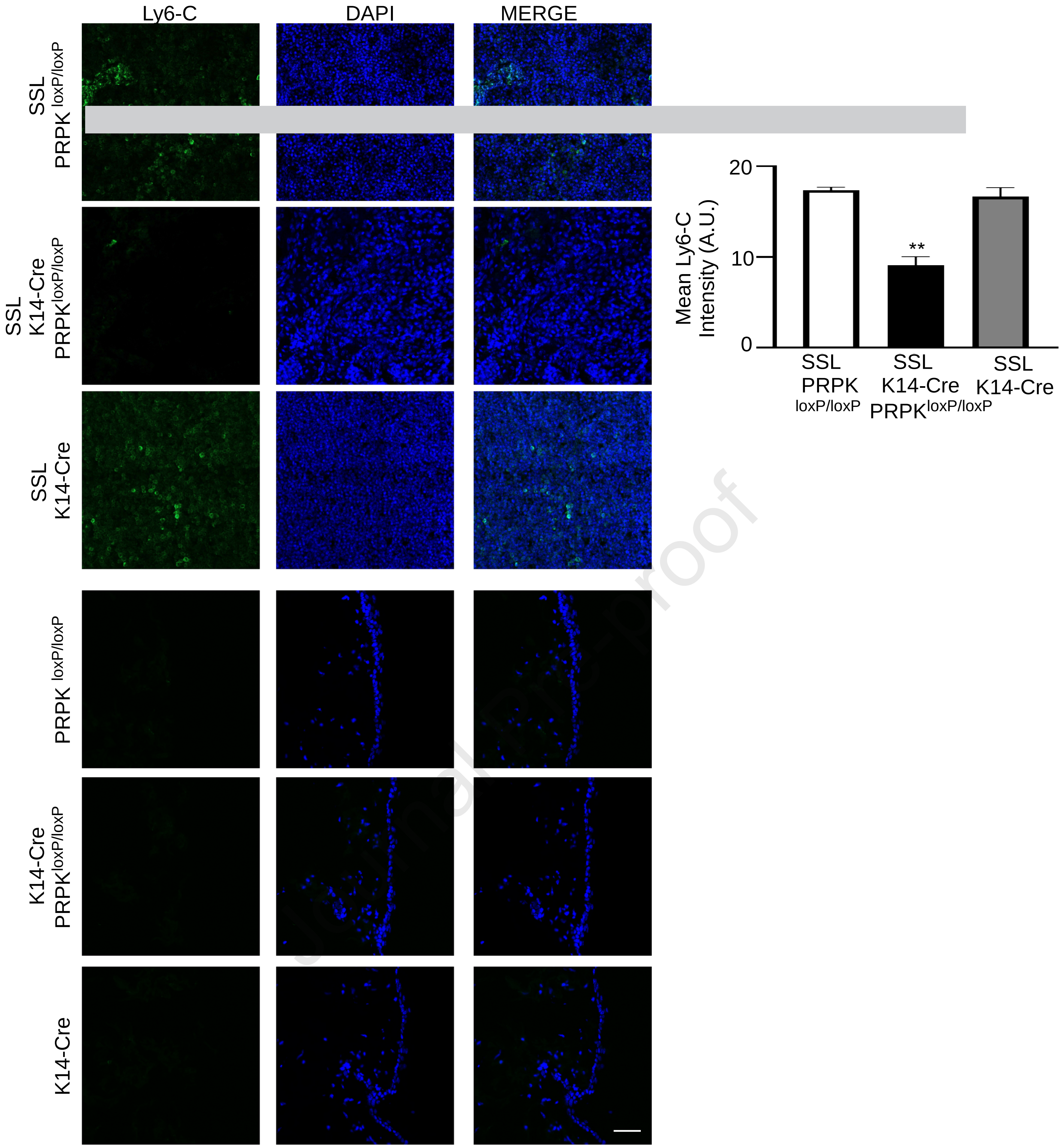

Journal Pre-proof

Supplementary Figure 9

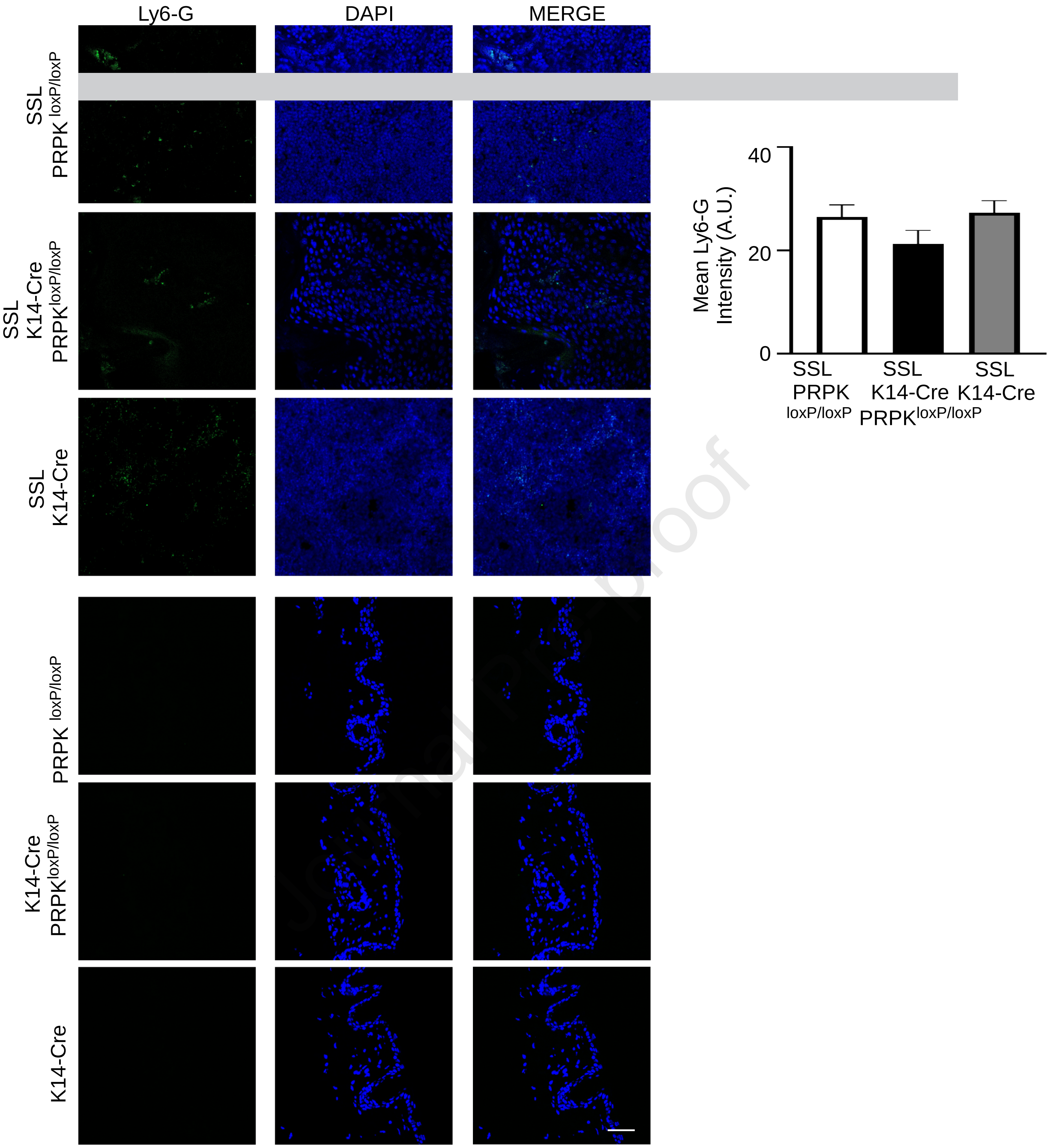

Journal Pre-proof

Supplementary Figure 10

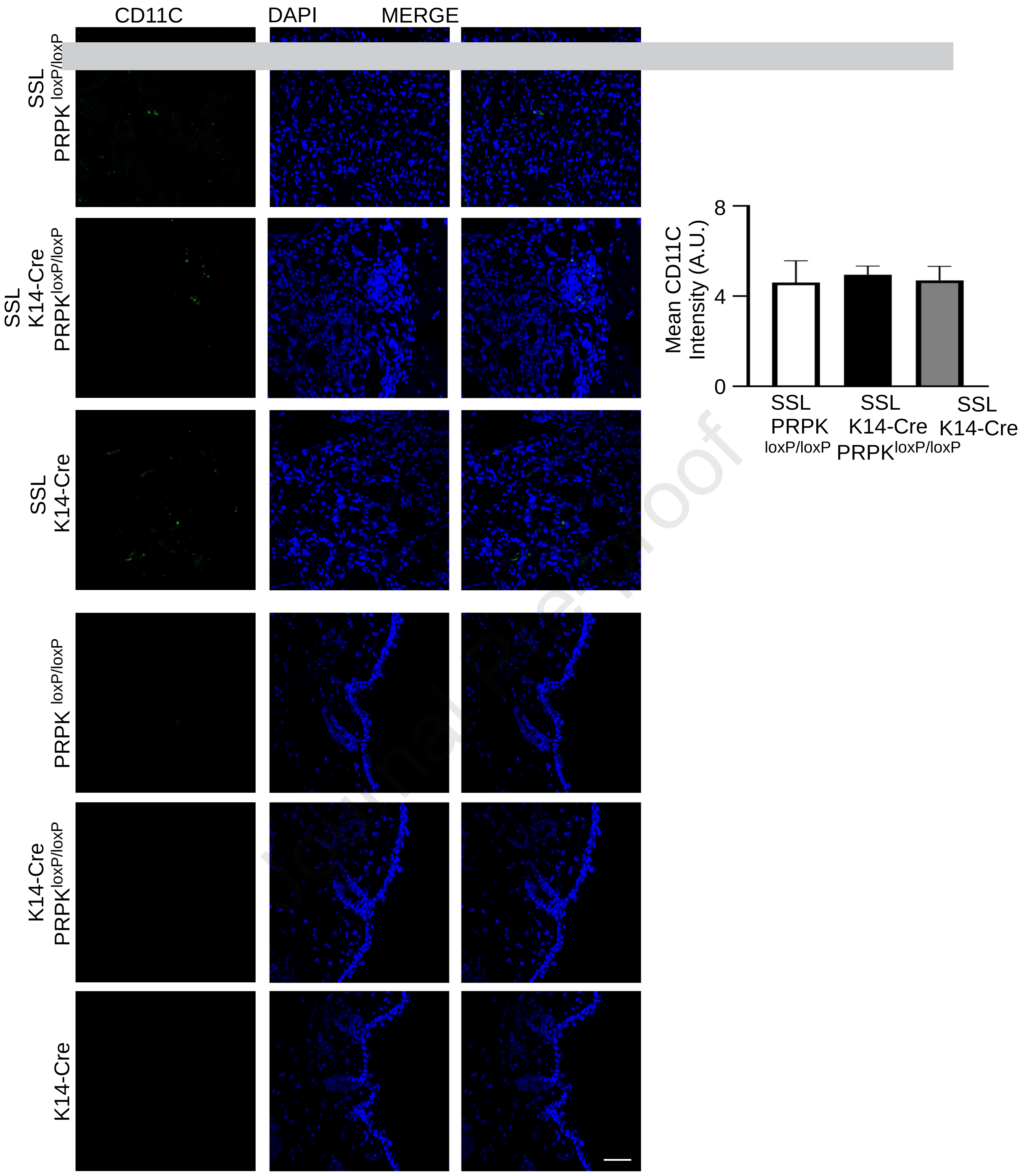

Journal Pre-proof
